# Ancient truncated FtsZ paralogs likely tune cell division in Hyphomicrobiales

**DOI:** 10.1093/g3journal/jkag275

**Published:** 2026-09-26

**Authors:** Brody Aubry, Amelia M Randich, Bailey Hudson, Elizabet Horton, Pamela J B Brown

**Affiliations:** Division of Biological Sciences, University of Missouri, 612 Hitt St., Columbia, MO 65201, United States; Department of Biology, University of Scranton, 800 Linden Street, Scranton, PA 18510, United States; Division of Biological Sciences, University of Missouri, 612 Hitt St., Columbia, MO 65201, United States; Division of Biological Sciences, University of Missouri, 612 Hitt St., Columbia, MO 65201, United States; Division of Biological Sciences, University of Missouri, 612 Hitt St., Columbia, MO 65201, United States

**Keywords:** FtsZ, gene duplication, paralogs, bacterial cell division

## Abstract

Bacterial cell division is a well-conserved, tightly regulated process that allows the separation of 2 viable daughter cells. In most bacteria, the proteins that drive division, termed the divisome, are recruited to mid-cell by FtsZ after it polymerizes to form the Z-ring. Interestingly, FtsZ has undergone several independent duplication events across the bacterial kingdom. In this work, we searched for FtsZ GTPase protein sequences within Alphaproteobacterial genomes and observed numerous *ftsZ* duplications in the order Hyphomicrobiales. Hidden Markov Modeling (HMM) supported the maintenance of 2 distinct lineages of FtsZ GTPase duplications among 3 families. The *Nitrobacteraceae* duplication, occurring in only the genus *Bradyrhizobium*, exhibits a different substitution pattern from that shared by the *Phyllobacteriaceae* and *Rhizobiaceae* families. Within the *Rhizobiaceae* lineage, *Agrobacterium tumefaciens* contains 3 paralogs of FtsZ including the essential FtsZ_AT_ and paralogs FtsZ_1_ and FtsZ_3_. We show that FtsZ_1_, but not FtsZ_3_, inhibits cell division when expressed continuously. A hyperactive allele of *ftsW* partially protected against continuous expression of *ftsZ_1_*, suggesting that FtsZ_1_ may function to negatively regulate septal peptidoglycan biosynthesis during cell division. Overall, these observations suggest that maintenance of FtsZ paralogs in some bacteria may help control the division process.

## Introduction

Nearly all bacteria depend on the ancient cytoskeletal scaffold protein FtsZ to enable cell division and produce the next generation of cells (Megrian et al. 2022). This protein forms the “Z-ring” and recruits a large complex of divisome proteins necessary to orchestrate cell division (Radler and Loose 2024). While most bacteria have a single copy of *ftsZ*, at least 5 independent duplications have been observed across the bacterial kingdom (Vaughan et al. 2004; Margolin 2005; Howell et al. 2019). Interestingly, these paralogs are often truncated and are not confined to one class of bacteria. Despite the distribution and frequency of *ftsZ* duplications, little is known about why these truncated *ftsZ* genes are maintained in genomes and if they encode novel functions. Here, we sought to expand our knowledge about the function of FtsZ paralogs within the Alphaproteobacterial class of bacteria.

Our understanding of FtsZ and its role in cell division is built on decades of work studying full-length, “canonical” *ftsZ* that is encoded in the division and cell wall (*dcw*) operon alongside other essential divisome components (Megrian et al. 2022). FtsZ monomers polymerize at mid-cell to form a ring of dynamic filaments that are anchored to the inner membrane. The Z-ring forms a scaffold for the recruitment of division proteins, forming the mature divisome. Collectively, the divisome components complete 4 major steps of bacterial cell division: formation/anchoring of the Z-ring, recruitment of divisome proteins, activation of septal peptidoglycan synthases (FtsW/FtsI), and separation of daughter cells (Santiago-Collazo et al. 2024). Full-length *ftsZ* sequences possess 4 structural domains: N-terminal peptide (NTP) region, GTPase domain, C-terminal linker (CTL), and C-terminal domain (CTD) (Fig. 1, gray box). The GTPase domain drives the dynamic polymerization mediated by the binding and hydrolysis of GTP (de Boer et al. 1992; Mukherjee and Lutkenhaus 1994; Erickson et al. 1996), the CTD is involved in anchoring (Addinall and Lutkenhaus 1996; Ma et al. 1996; Wang et al. 1997), and the CTL has roles in protein interactions and divisome regulation (Buske and Levin 2013; Huecas et al. 2017; Sundararajan and Goley 2017; Barrows et al. 2020). Lastly, the NTP, although more variable and less understood, has been shown to interact with regulating partners (Huang et al. 1996; Tan et al. 2011; Corrales-Guerrero et al. 2018).

**Fig. 1.**
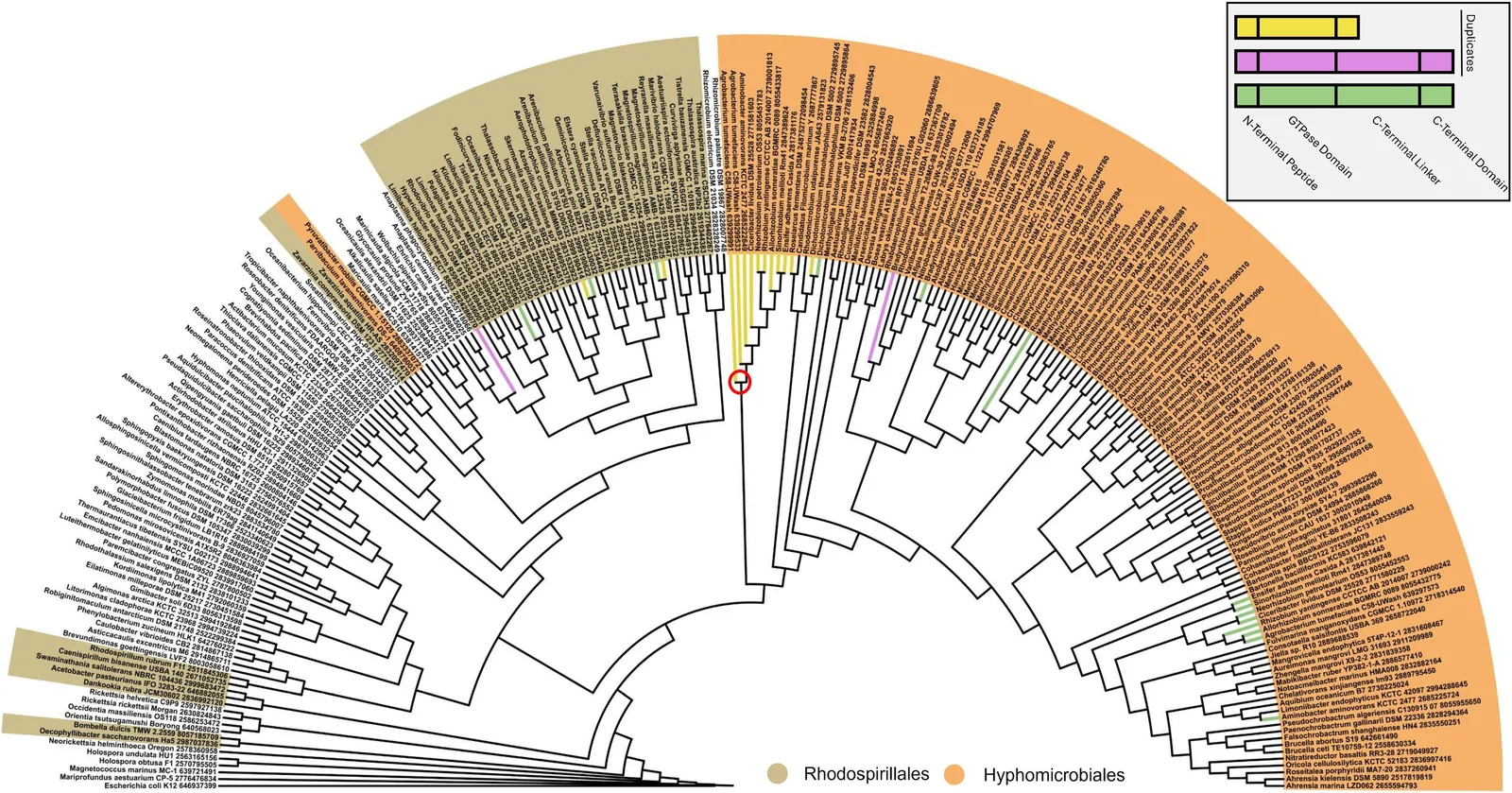
FtsZ duplications have arisen multiple times in Alphaproteobacterial class. GTPase domain tree of select Alphaproteobacteria reveals gene duplications across several orders of Alphaproteobacteria. Branches in black indicate that species that have a single copy of FtsZ. Colored branches indicate species with FtsZ duplications. Green branches lead to canonical FtsZ while yellow and purple branches indicate duplicated FtsZ paralogs. Representative domain structures for canonical (green) and duplicate (yellow & purple) FtsZ copies are shown in the gray box at the top-right. *Bradyrhizobium japonicum* is the only duplicate in the Hyphomicrobiales order that possesses all 4 structural motifs for FtsZ. Red circle indicates a node comprised solely of duplicated FtsZ paralogs. Color-coded names indicate the Alphaproteobacterial order of the organisms containing the FtsZ protein.

Duplicate *ftsZ* sequences are found outside of the *dcw* operon, and most of them are truncated and missing one or more of the C-terminal motifs (Vaughan et al. 2004). Within the Alphaproteobacterial class, Vaughan et al. described 6 duplicates containing NTP, GTPase domain, and partial CTL motifs. Since this description, genome sequencing efforts have extended the available dataset across the Alphaproteobacterial class. In this work, we expanded the analysis of *ftsZ* duplications to each described genus in the Alphaproteobacterial class as well as 831 species within the Hyphomicrobiales order. This analysis provides further evidence that 2 independent *ftsZ* duplications occurred within the Hyphomicrobiales order of the Alphaproteobacterial class, resulting in distinct *Rhizobiaceae*/*Phyllobacteriaceae* and *Nitrobacteraceae* lineages. Mapping the unique substitution patterns in these 2 groups to structural models suggests positive selection for functions distinct from canonical FtsZ. We explored possible functions of the *ftsZ* duplications in the *Rhizobiaceae*/*Phyllobacteriaceae* group by manipulating *ftsZ*_1_ and *ftsZ*_3_ genes in *Agrobacterium tumefaciens*. Continuous expression of FtsZ_1_ resulted in a complete block in cell division and morphological defects that were dependent on its enzymatic activity. This strain produced large, round cells indicative of misregulation of peptidoglycan biosynthesis. Consistent with this possibility, we found that a hyperactive allele of *ftsW*, the monofunctional transglycosylase for septal peptidoglycan synthesis, partially protected against constitutive FtsZ_1_ expression. Overall, these results indicate that the truncated FtsZ_1_ paralog in *A. tumefaciens* contributes to regulation of division processes such as septal peptidoglycan synthesis.

## Materials and methods

### Bacterial strains, plasmids, and growth conditions

All bacterial strains and plasmids used in this study are provided in the Strain List found in Supplementary File 2. *A. tumefaciens* was grown in *A. tumefaciens* glucose and nitrogen (ATGN) minimal media at 28 °C with shaking (Tempé et al. 1977; Morton and Fuqua 2012). For plasmid selection, kanamycin was used at a working concentration of 300 µg/mL for *A. tumefaciens.* When culturing *Escherichia coli*, lysogeny broth (LB) medium was used, and cells were grown at 37 °C with shaking (Bertani 1951). When appropriate, 50 µg/mL of kanamycin was added.

For spotting assays, indicated media were prepared with 1.5% agar. The agar plates included ATGN minimal media in the main-text figures as well as the following media for Supplementary Fig. 4 in Supplementary File 1: ATSN minimal media, LB, and LB0 (which follows the same recipe as LB without NaCl). When appropriate, ATGN was supplemented with ampicillin, cephalexin, and aztreonam to a final concentration of 10 µg/mL, cefsulodin to 25 µg/mL, acetosyringone to 200 µM, kanamycin to 300 µg/mL, or 1 mM isopropyl β-D-1-thiogalactopyranoside (IPTG).

### Construction of plasmids and strains

FtsZ_1_ and FtsZ_3_ were PCR amplified with 20 base pairs of overlap, with regard to the amplified pSRKKM Ptac backbone, using primers listed in the Plasmid & Primer List in Supplementary File 2. The PCR products were joined using Gibson Assembly to construct the plasmids listed in the Plasmid & Primer List in Supplementary File 2. Each plasmid was transformed into *E. coli* DH5α strains following the New England Biolabs standard protocol for heat-shock transformations. Plasmids were maintained in *E. coli* DH5α and were miniprepped and sequence-verified (Plasmidsaurus). Successful constructs were electroporated into *E. coli* S17-1 λpir, which was subsequently used to transfer the plasmid into *A. tumefaciens* wild type or FtsW F137L (FtsW*) backgrounds. The mating procedure occurs by mixing cell pellets of overnight S17-1 λpir+ vector and *A. tumefaciens* cultures, each resuspended in 50 µL and placed onto sterile filter paper on LB IPTG plates. After 24 h of incubation at 28 °C, cells were collected and serially diluted onto ATGN Kan 300 µg/mL plates: which *E. coli* cannot grow on.

### Alphaproteobacterial FtsZ duplication phylogenetic tree construction

Using the Joint Genome Institute (JGI) database, at least one representative from each genus within the Alphaproteobacteria was identified (Grigoriev et al. 2012; Nordberg et al. 2014). Within this set of genera, we prioritized those with genomes that were finished or permanent drafts. The embedded NCBI protein–protein BLAST function was used to identify FtsZ copies within each genome (Altschul et al. 1990). First, we conducted a BLAST search with FtsZ_AT_ from *A. tumefaciens.* We repeated BLAST searches using FtsZ sequences from *Sphingosinicella microcystinivorans*, *Anaplasma phagocytophilum*, *Ehrlichia canis*, *Magnetococcus marinus*, and FtsZ_1_ from *A. tumefaciens* as a query. No additional FtsZ sequences were identified with the additional BLAST searches, suggesting that our searches identified all *ftsZ* paralogs within these genera.

Once the FtsZ sequences were identified, MEGA11 was used to align all FtsZ sequences with the embedded Clustal alignment tool (Tamura et al. 2021). Then, FtsZ_AT_ was used as a guide to trim all sequences down to the GTPase domain. Finally, the best model for substitutions was estimated to be LG + G + I using MEGA11. Next, we uploaded the aligned, truncated FtsZ file to BEAST v1.10.4, and used the substitution model LG + G + I. For the “Tree Prior” setting, we selected Speciation: Yule Process (Yang 1994; Gernhard 2008; Le and Gascuel 2008; Drummond et al. 2012; Suchard et al. 2018). When appropriate, the seed 1708101569949 was used. For the MCMC settings, the length of chain was set to 10,000,000 with screening every 1,000. After running through the 10,000,000 iterations on BEAST, the maximum clade credibility tree was constructed with TreeAnnotator: part of the BEAST package. We followed the default suggestion of using 10% of all states calculated: 1,000,000. The Maximum Clade Credibility tree was visualized with iTOL (Letunic and Bork 2021). Copies found in the *dcw* operon were identified as the canonical FtsZ copy using the built-in genome viewer of JGI/IMG (Grigoriev et al. 2012; Nordberg et al. 2014).

### Cell viability spotting assay

Cells were inoculated into 1 mL of ATGN and incubated for overnight growth of 16 to 21 h. Each strain was diluted into a new culture tube to result in an OD_600_ = 0.1. Afterwards, 4 to 6 h of outgrowth allowed for cultures to reach exponential phase growth (OD_600_ = 0.4 to 0.6). Each strain was then diluted to OD_600_ = 0.1 before being serially diluted in 10-fold increments from series 10^0^ through 10^−7^. After diluting, 3 μL of each dilution was pipetted onto 1.5% ATGN agar plates. Plates were allowed to dry for 1 h at room temperature and then were transferred to 28 °C for 72 h. Plates were imaged with the Bio-Rad ChemiDoc MP imager.

### Minimal inhibitory concentration analysis

To prepare for antimicrobial minimum inhibitory concentration (MIC) analyses, strains were grown to exponential phase as described for the viability assay. Subsequently, cells were diluted to OD_600_ = 0.125 and used to saturate a sterile cotton swab. The saturated cotton swab was used to lawn cultures onto an ATGN plate. MIC strips were placed in the center of the plate using sterile forceps. Plates were incubated for 2 d at 28 °C and then imaged.

### Microscopy and quantitative image analysis

To prepare for microscopy, strains were grown to exponential phase as described for the viability assay. For phase microscopy, 0.5 μL of cells were spotted onto 1.75% ATGN agarose pads and imaged using an inverted Nikon Eclipse TiE with a QImaging Rolera EM-C2 1K EMCCD or pco.edge 4.2 sCMOS camera and Nikon Elements Imaging Software. For expression experiments, 1 mM IPTG was added to the liquid medium and bacterial populations were imaged by spotting the cultures onto pads at specific time points. For time-lapse microscopy, 1 mM IPTG was instead added to agarose pads and images were acquired every 10 min for a set field of views. Phase microscopy images were converted to binary masks using ImageJ (Rueden et al. 2017). Binary masks were processed using CellTool to measure the area of each cell outline following the CellTool tutorial (Pincus and Theriot 2007). Area measurements were visualized using R-Studio (The R Core Team 2023). For HADA-DA images, cells were co-incubated with 0.2 mM HADA-DA and 1 mM IPTG in liquid for 5 h and 45 min. Subsequently, cells were pelleted by 7,000 rpm for 5 min and resuspended in ATGN 3 times before being fixed with 1 mL of ice-cold 70% ethanol, at −20 °C for 15 min, before a final pelleting and resuspension in PBS.

### Hidden Markov and PyMOL modeling

FtsZ and FtsZ GTPase duplications were identified and collected from genomes on JGI, as described above, but every genome was selected within the Hyphomicrobiales order shared between JGI and the List of Prokaryotic names with Standing in Nomenclature (Supplementary Table 2, Supplementary File 2; Parte et al. 2020). Protein sequences from *Nitrobacteraceae* (canonical *n* = 36, duplicate *n* = 38), *Phyllobacteriaceae* (canonical *n* = 80, duplicate *n* = 84), and *Rhizobiaceae* (canonical *n* = 142, duplicate *n* = 157) families were aligned to FtsZ GTPase domains from *E. coli* (IMG ID 2978828590) and *A. tumefaciens* (NP_532761.1) as separate groups using Jalview (Troshin et al. 2011). The GTPase domain was defined as the first 315 amino acids of the *E. coli* FtsZ GTPase domain. Only sequences with unambiguous alignment and full coverage of the GTPase domain were included in this analysis. When necessary, the *N*-termini of sequences were trimmed to the positions of the conserved 15 amino acids of canonical FtsZ from Hyphomicrobiales NTP (Fig. 3b). WebLogo 3 was used to plot the amino acid distribution at each position of the NTP and GTPase domains (Suchard et al. 2018). Structural models were generated with PyMol using 6UNX (PDB) as a scaffold and color-coding the conservation patterns using the HMMs that maintained *E. coli* amino acid numbering (The PyMOL Molecular Graphics System, Version 1.2r3pre, Schrödinger, LLC.).

## Results and discussion

### Duplicate FtsZ proteins are distributed throughout the Hyphomicrobiales order

Several FtsZ duplicates were identified previously within the Alphaproteobacterial class including those found in *A. tumefaciens*, *S. meliloti*, *B. japonicum*, and *Magnetospirillum magneticum* (Vaughan et al. 2004). To better understand the distribution and function of these FtsZ duplicates, we took advantage of the rapidly growing number of publicly available whole-genome sequences to generate an FtsZ GTPase domain tree using representatives from each genus across the Alphaproteobacterial class (Fig. 1; Supplementary Fig. 1, Supplementary File 1). FtsZ protein sequences were identified through iterative NCBI BLAST using the FtsZ sequences from *A. tumefaciens*, *S. microcystinivorans*, *E. canis*, *M. marinus*, and the FtsZ_1_ sequence from *A. tumefaciens* (Supplementary Table 1, Supplementary File 2). Each BLAST iteration resulted in the same results, indicating that the identified FtsZ duplications share enough conservation with the canonical FtsZ sequences to allow differentiation from other GTPase domain-containing genes. For species that possessed multiple hits, the FtsZ copy encoded in the *dcw* operon was identified as the canonical sequence while those outside of this operon were classified as duplicates.

Known duplications across *A. tumefaciens*, *S. meliloti*, and *B. japonicum* were observed (Vaughan et al. 2004), as well as previously unidentified FtsZ duplicates in *Limibacillus halophilus*, *Arenibaculum pallidiluteum*, *Aminobacter aminovorans*, *Ciceribacter lividus*, *Neorhizobium petrolearium*, *Rhizobium yantingense*, *Allorhizobium sonneratiae*, *Ensifer adhaerens*, *Roseiarcus fermentans*, and *Dichotomicrobium thermohalophilum* (Fig. 1; Supplementary Fig. 1, Supplementary File 1). Notably, the duplicate identified in *L. halophilus*, as well as the previously described duplicate in *B. japonicum*, were the only duplicates that possessed a CTD while all other duplicates ended with GTPase domains or minimal CTLs (Fig. 1, inset gray box). Furthermore, *A. tumefaciens* is the only species that possesses 2 duplicate FtsZ alleles in Fig. 1.

Two patterns of FtsZ duplications were readily observed: first, some FtsZ duplicates were most closely related to the canonical FtsZ GTPase domain from the same organism. Examples of this pattern were observed in the Rhodospirillales order (*A. pallidiluterum* and *M. magneticum*) as well as the Hyphomicrobiales order (*D. thermohalophilum*) (Fig. 1; see pairs of yellow and green branches; Supplementary Fig. 1, Supplementary File 1). This pattern is consistent with recent gene duplications giving rise to the secondary copies of FtsZ within each species. In the second pattern, the majority of the identified FtsZ duplicates were instead more closely related to duplicate FtsZ GTPase domains from other species than to the canonical FtsZ in the same species. A clade comprised solely of FtsZ duplicates in the Hyphomicrobiales order consisted of *A. tumefaciens*, *A. aminovorans*, *C. lividus*, *N. petrolearium*, *R. yantigense*, *A. sonneratiae*, *S. meliloti*, and *E. adhaerens* (marked by a red circle at the base of the clade in Fig. 1 and Supplementary Fig. 1, Supplementary File 1). The formation of this clade within the *Rhizobiaceae* and *Phyllobacteriaceae* families suggests that these FtsZ GTPase duplications are potentially orthologs of each other with a distinct evolutionary trajectory from canonical FtsZ GTPase domains. Vaughan et al. also identified duplications in each of these families and our observations are consistent (Vaughan et al. 2004).

To better understand FtsZ duplication patterns within the Hyphomicrobiales, we completed a fine-scale analysis of the distribution of FtsZ duplicates within this order. Consistent with observations in Fig. 1, we found that FtsZ duplications within *Roseiarcaceae* and *Hyphomicrobiaceae* are largely constrained to 3 families (Fig. 2; Supplementary Table 2, Supplementary File 2), with a few additional duplicates arising in other families. In the *Hyphomicrobiaceae,* the duplicate identified in *D. thermohalophilum* paired with the canonical sequence, suggesting a recent event (Fig. 1; Supplementary Fig. 1, Supplementary File 1). Interestingly, the duplicate FtsZ GTPase domain from the *Roseiarcaceae* member, *R. fermentans*, did not cluster with the duplicate clade in Fig. 1 or its respective canonical sequence. However, these observations could be due to the relatively limited number of genome sequences published for these families and further analysis, as new data becomes available, will be necessary.

**Fig. 2.**
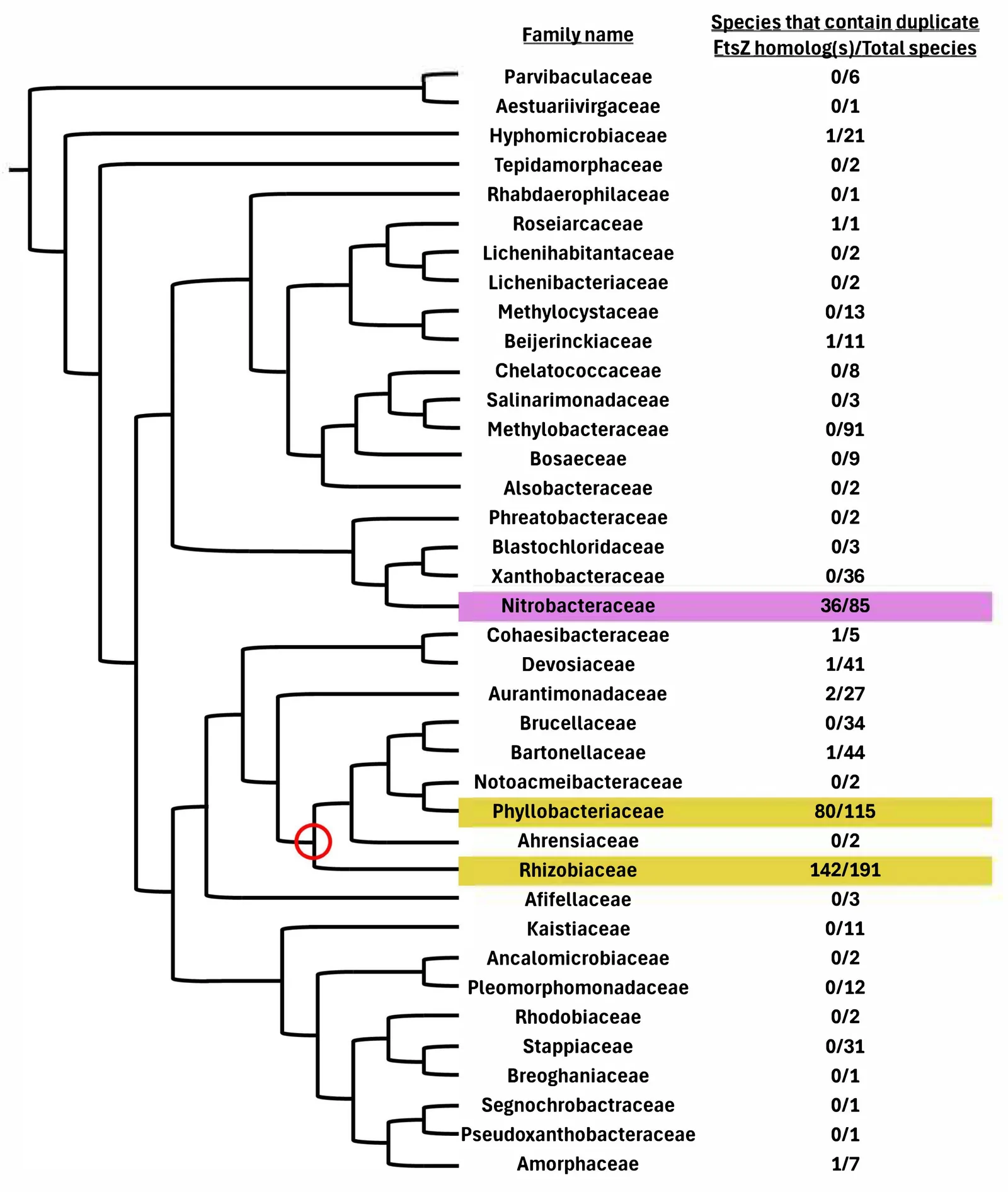
FtsZ duplications are present, primarily, in 3 families of the Hyphomicrobiales order. a) Using the Joint Genome Institute/Integrated Microbial Genomes & Microbiomes database, each species detailed within the Hyphomicrobiales order, as annotated by the List of Prokaryotic names with Standing in Nomenclature, was assessed for the presence or absence of FtsZ duplications with protein similarity searches and synteny comparisons. FtsZ sequences identified were grouped by family without regard of multiple duplications. Cladogram was drawn with no evolutionary data but was based off previous work (diCenzo et al. 2024). The circle indicates the last shared node between Rhizobiaceae and Phyllobacteriaceae families.

Retention of a truncated FtsZ duplicate occurs at a very high frequency (∼70%) in the *Rhizobiaceae* and *Phyllobacteriaceae* families (Fig. 2; Supplementary Table 2, Supplementary File 2). These duplicates cluster together and therefore are more closely related to each other than to the canonical FtsZ in their cognate species (Fig. 1; Supplementary Fig. 1, Supplementary File 1). This pattern is consistent with an ancient acquisition of a duplicate FtsZ GTPase prior to the divergence of these families. We identified another FtsZ duplication within the *Nitrobacteraceae,* with over 40% of the sequenced species retaining the duplicate, but this was constrained to the genus *Bradyrhizobium* (Fig. 2; Supplementary Table 2, Supplementary File 2). Therefore, the size of this cluster of duplications is due to the extensive sampling of this genus for genomic sequencing. Moreover, all *Bradyrhizobium* duplicates possessed the 4 canonical FtsZ domains. This contrasts with the *Rhizobiaceae* and *Phyllobacteriaceae* duplications where the duplicates are truncated to the NTP, GTPase domain, and short CTLs (Fig. 1, upper right inset). Hereafter, we refer to the *Nitrobacteraceae* duplicates as the *Bradyrhizobium* duplicates since all duplications are found within that genus. Based on these observations, we next used a bioinformatic approach to explore the similarities and differences between the *Rhizobiaceae*, *Phyllobacteriaceae*, and *Bradyrhizobium* canonical and duplicate FtsZ GTPases.

### Two independent FtsZ GTPase duplications have arisen and have been maintained in the Hyphomicrobiales order

To understand the relationship of the Hyphomicrobiales duplicate FtsZ proteins with their canonical FtsZ counterparts and with each other, we trimmed canonical and duplicate FtsZ sequences down to the GTPase domain and created amino acid consensus sequences (Fig. 3a; Supplementary Fig. 2, Supplementary File 1). Because we expected to see strict conservation of residues required for the function of the domain, comparison of these consensus sequences could pinpoint which regions of the GTPase domain are under purifying selection in the duplicates. Residues with high conservation in the duplicate FtsZ GTPases that are chemically distinct from conserved residues in the canonical FtsZ GTPase domain could be under selection for new function. In contrast, positions with high variability in the duplicate FtsZ GTPase that are strictly conserved in the canonical FtsZ GTPase domain are no longer under selection for the original structure and/or function of the FtsZ GTPase domain. Identifying regions with these patterns would suggest which portions of the duplicate FtsZ GTPase domain have been remodeled for a function distinct from that of the canonical FtsZ protein.

**Fig. 3.**
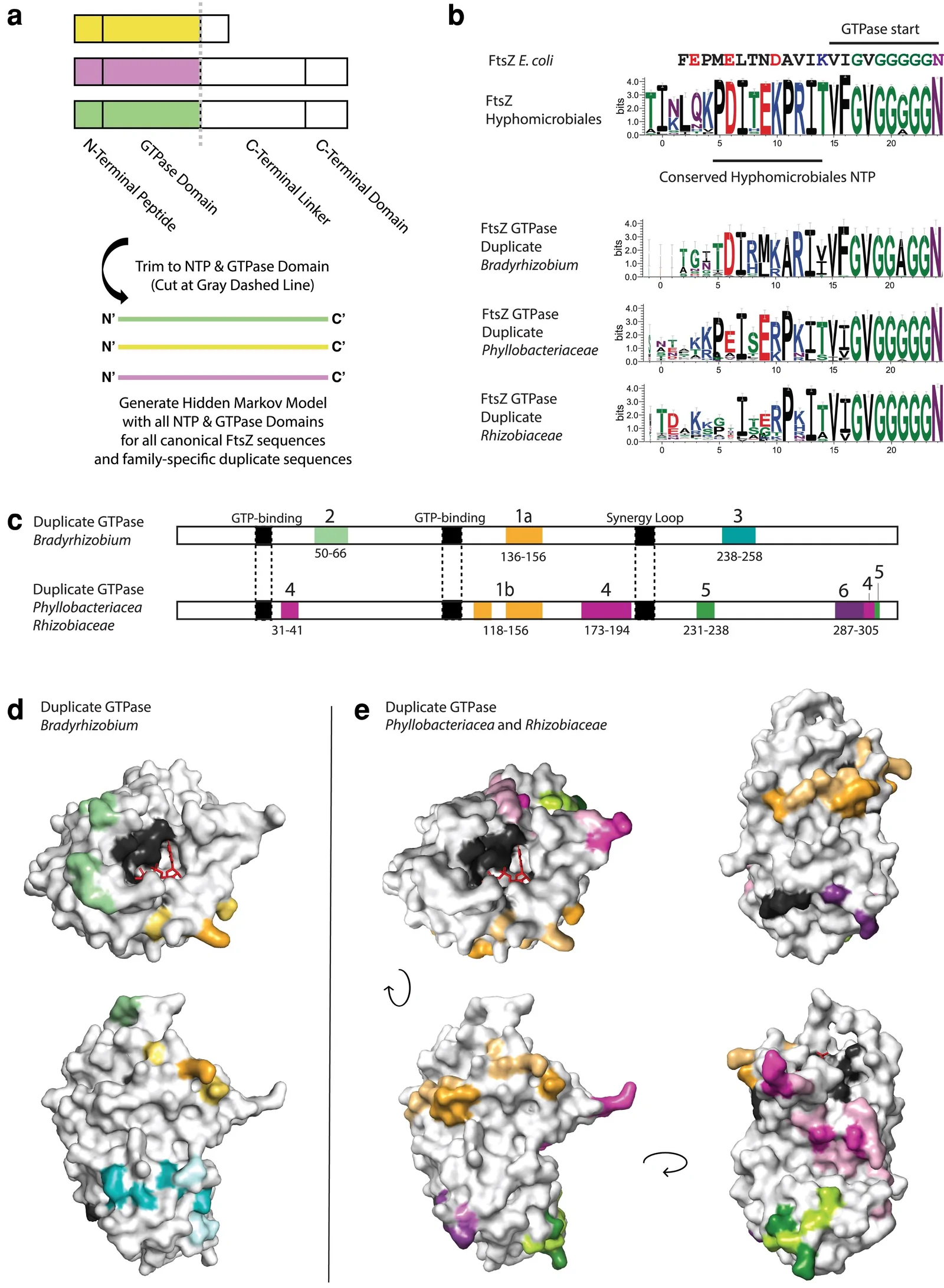
FtsZ GTPase duplicates are conserved within 3 Hyphomicrobiales families and constitute 2 distinct lineages. a) Schematics of FtsZ and its paralogous GTPase domain duplications and explanation of approach. b) Hidden Markov Model (HMM) logos indicating the consensus protein sequences of the leader regions of Hyphomicrobiales FtsZ (*n* = 260 sequences) and the duplications identified in the *Bradyrhizobium* (*n* = 38 sequences), *Phyllobacteriaceae* (*n* = 82 sequences), and *Rhizobiaceae* families (*n* = 152 sequences) using WebLogo 3 (Suchard et al. 2018). Amino acids are color coded according to chemical properties, with uncharged polar residues in green, neutral residues in purple, basic residues in blue, acidic residues in red, and hydrophobic residues in black. The height of each letter is proportional to the relative frequency of a given identity, and the height of the stack indicates the sequence conservation at that position. *E. coli* FtsZ numbering is used for ease of comparison. c) Schematic summary of HMM analysis of the entire duplicated GTPase domains. Because *Phyllobacteriaceae* and *Rhizobiaceae* share the same HMM patterns, they are shown as one schematic. Black regions are conserved regions that form the GTP-binding catalytic site (Löwe and Amos 1998) and have been shown to be involved in GTP hydrolysis (Lu et al. 2001). Dashed lines between the 2 sequences indicate conservation across all 3 families. Numbered, colored boxes indicate remodeled regions of interest that have been mapped to the structural models in d) and e). Full-length HMMs are provided in Supplementary Fig. 2, Supplementary File 1. Numbering is based on *E. coli* FtsZ. d) Remodeled regions from the HHM of the *Bradyrhizobium* GTPase duplicate mapped onto *E. coli* FtsZ (PDB 6UNX). Colored regions correspond to the remodeled regions indicated in (c) with dark shading indicating a conserved substitution with a chemical change from canonical FtsZ GTPase and light shading indicating loss of conservation from canonical FtsZ GTPase (see Supplementary Fig. 2, Supplementary File 1 for HHM and residue identities). GTP is shown in red. e) Remodeled regions from the HHM of the *Phyllobacteriaceae* and *Rhizobiaceae* GTPase duplicate, as in d).

As expected, the canonical FtsZ GTPase domains exhibit high conservation with the *E. coli* FtsZ GTPase domain except for the NTP (Fig. 3b). The NTP has extensive variation across the bacterial domain, varying from as few as 6 residues in some Firmicutes to as many as 77 residues in Actinomycetes (Vaughan et al. 2004). This variation could underlie family-specific differences with interacting regulatory partners. For instance, the first 32 residues of *E. coli* FtsZ have been reported to interact with FtsZ-inhibiting proteins YeeV (Tan et al. 2011) and SulA (Huang et al. 1996). Furthermore, the conserved 51 residue NTP of heterocyst-forming cyanobacteria has been shown to contribute to the polymerization behavior and binding interactions with the essential divisome component SepF in *Anabaena sp. PCC 7120* (Corrales-Guerrero et al. 2018). This region ranges from 7 to 44 amino acids in the Alphaproteobacteria (Vaughan et al. 2004), although no regulatory function has been reported for any members. The portion of the NTP proximal to the start of the GTPase domain, PDITEKPR, is invariant among all the canonical Hyphomicrobiales sequences with the preceding 6 N-terminal residues exhibiting general conservation with some substitutions (Fig. 3b). The strict maintenance of this region within this order suggests that it may behave as a scaffold for protein–protein interactions.

In comparison to the strict conservation in the canonical FtsZ sequences, the duplicate NTPs exhibit diminishing conservation by sequence identity or similarity (Fig. 3b). In particular, the first 6 or 7 residues are highly variable, constituting the largest remodeled region in all the HMMs (Supplementary Fig. 2, Supplementary File 1), followed by mostly conservative substitutions in the PDITEKPR sequence. The duplicate FtsZ GTPases from the *Rhizobiaceae* family exhibit the most variation and loss of conservation in the NTP. Overall, the loss of strict conservation of the NTP in the duplicate FtsZ GTPase domains may indicate that this region is under different evolutionary constraints than the NTP of canonical FtsZ. Thus, we hypothesize that the canonical and duplicate FtsZ proteins are not regulated at the *N*-terminus by the same regulatory networks in the *Rhizobiaceae* and *Phyllobacteriaceae* families.

In stark contrast to the variation seen in the NTP region, the remainder of the duplicate FtsZ GTPase domains strictly conserve the GTP-binding pocket and other residues shown to influence GTPase activity (Fig. 3c black boxes and Fig. 3d and e black residues; Supplementary Fig. 2, Supplementary File 1; Löwe and Amos 1998; Lu et al. 2001). Moreover, residues in the GTPase domain predicted to bind regulators FzlA and MipZ are also highly conserved (Supplementary Fig. 3, Supplementary File 1; Corrales-Guerrero et al. 2022; Payne et al. 2024). This suggests that the duplicate GTPases are still active and could overlap in function with the canonical FtsZ GTPase domain in the Z-ring. Indeed, in *A. tumefaciens,* duplicate FtsZ_1_ likely forms functional co-polymers with FtsZ_AT_ (Howell et al. 2019). Previous work has shown that the co-polymers form similar structures and maintain GTP hydrolysis rates comparable to homopolymers of FtsZ_AT_ in vitro (Howell et al. 2019). In vivo, localization of FtsZ_1_ to the mid-cell is dependent on the presence of FtsZ_AT_ which suggests that FtsZ_1_ cannot form homopolymers (Howell et al. 2019). These observations indicate that the FtsZ duplicates may regulate cell division when incorporated into the Z-ring. It is intriguing to speculate that the conditional formation of FtsZ co-polymers may tune cell division by promoting or inhibiting Z-ring dynamics in response to differing environmental conditions.

When compared to the canonical FtsZ consensus sequence, the consensus sequences of the duplicate FtsZ GTPase domains from *Phyllobacteriaceae* and *Rhizobiaceae* share the same remodeling pattern (Fig. 3c; Supplementary Fig. 2, Supplementary File 1), whereas the duplicate FtsZ GTPase domain from the *Bradyrhizobium* group exhibits a distinctly different pattern with fewer substitutions. We mapped these positions to structural models of the *E. coli* FtsZ GTPase domain to understand their spatial relationship to each other and to conserved regions known to interact with GTP (Fig. 3d and e) or regulatory proteins FzlA and MipZ (Supplementary Fig. 3, Supplementary File 1). Figure 3c summarizes 6 regions of denser remodeling of proximal surface residues to illustrate the 2 different substitution patterns (see Supplementary Fig. 2, Supplementary File 1 for the entire consensus sequences with identified regions). These remodeled regions were defined both by positions with invariant substitutions that change the chemical nature of the residue (darker hue) and positions with increased variation or relaxed conservation (lighter hue). The remodeling of these regions could modify the behavior of this duplicate FtsZ GTPase domain from the canonical FtsZ GTPase domain by facilitating interactions with different binding partners.

Overall, the remodeling patterns support the hypothesis that the *Phyllobacteriaceae* and *Rhizobiaceae* duplicate FtsZ proteins share a common ancestor, while the *Bradyrhizobium* duplication is an independent, more recent event. The lower number of substitutions in the *Bradyrhizobium* duplicate consensus sequence is expected for a duplication occurring more recently within a single genus. The clear split between the 2 lineages of duplicate FtsZ proteins agrees with the known phylogenetic relationships of these families (Fig. 2) and previous studies with fewer genomes (Vaughan et al. 2004). However, this observation also suggests that the duplication, while maintained in the *Phyllobacteriaceae* and *Rhizobiaceae* families, has been lost in 4 other Hyphomicrobiales families that share the same last common ancestor (Fig. 2, red circle). This surprising lack of parsimony could be explained by the extensive lifestyle diversity exhibited by the Hyphomicrobiales order (Williams et al. 2022a). Because the *Phyllobacteriaceae* and *Rhizobiaceae* families are known for soil-based lifestyles and plant interactions, while *Brucella* and *Bartonella* are families of animal pathogens, we wondered if the lifestyles of the families lacking the duplicate FtsZ GTPase were distinctly different. Within the families *Ahrensiaceae* and *Notoacmeibacteraceae*, we found that most species were originally isolated from marine environments (Supplementary Table 3, Supplementary File 2; Jung et al. 2012; Liu et al. 2016; Huang et al. 2017; Yan and Tuo 2019). Likewise, for genera that have lost the duplication in the *Phyllobacteriaceae* and *Rhizobiaceae* families, most were also isolated from marine environments or hosts (Supplementary Table 3, Supplementary File 2; Roh et al. 2008; Kämpfer et al. 2015; Li et al. 2016; Hyeon et al. 2017; Tóth et al. 2017; Cao et al. 2020; Song et al. 2023; Wang et al. 2023). Together these observations support the possibility that an ancient duplication of FtsZ has been maintained in the *Phyllobacteriaceae* and *Rhizobiaceae* lineages as an adaptation for specific terrestrial plant-associated or soil lifestyles. It is possible that the duplication in *Bradyrhizobium* arose convergently under the similar selective pressures; however, its distinct substitution pattern and limited distribution within a single genus make it challenging to speculate that the duplicates share similar functions.

### FtsZ_1_ production inhibits cell division

Overall, the maintenance of the duplicate *ftsZ* within the *Rhizobiaceae* and *Phyllobacteriaceae* lineages suggests that the duplicate FtsZ proteins may be under selection for a distinct function, such as an alternative GTPase in the Z-ring to adapt to stresses experienced in the soil environment. We used *A. tumefaciens* as a model to explore possible functions of FtsZ duplicates in a soil-dwelling microbe with the ability to invade a plant host. *A. tumefaciens* possesses 2 *ftsZ* duplicates, *ftsZ_1_* and *ftsZ_3_*, that are nonessential in standard laboratory conditions (Howell et al. 2019). Here, we exposed deletion mutants to a larger array of conditions, including host invasion and virulence. *ftsZ_1_* and *ftsZ_3_* were dispensable for viability in all tested conditions (Supplementary Figs. 4 and 5, Supplementary File 1). These observations are consistent with the finding that neither *ftsZ_1_* nor *ftsZ*_3_ are vital fitness factors for survival in tomato tumor, tomato root, and poplar root environments in TnSeq studies (Torres et al. 2022).

Next, we hypothesized that FtsZ duplicates may function as negative regulators of cell division. To test this hypothesis, we introduced a plasmid with the *ftsZ_1_* or *ftsZ_3_* gene under the control of the inducible *tac* promoter. In the presence of IPTG, we expected both FtsZ_1_ and FtsZ_3_ to accumulate in cells; however, only the stable accumulation of FtsZ_1_ was confirmed (Supplementary Fig. 6, Supplementary File 1). Accumulation of FtsZ_1_ caused a significant, 5-log decrease in viability (Fig. 4a). In contrast, FtsZ_3_ production was not confirmed and inducing conditions did not impair viability or morphology (Supplementary Fig. 6, Supplementary File 1). Thus, subsequent studies focused only on the phenotypes associated with plasmid-driven FtsZ_1_ production.

**Fig. 4.**
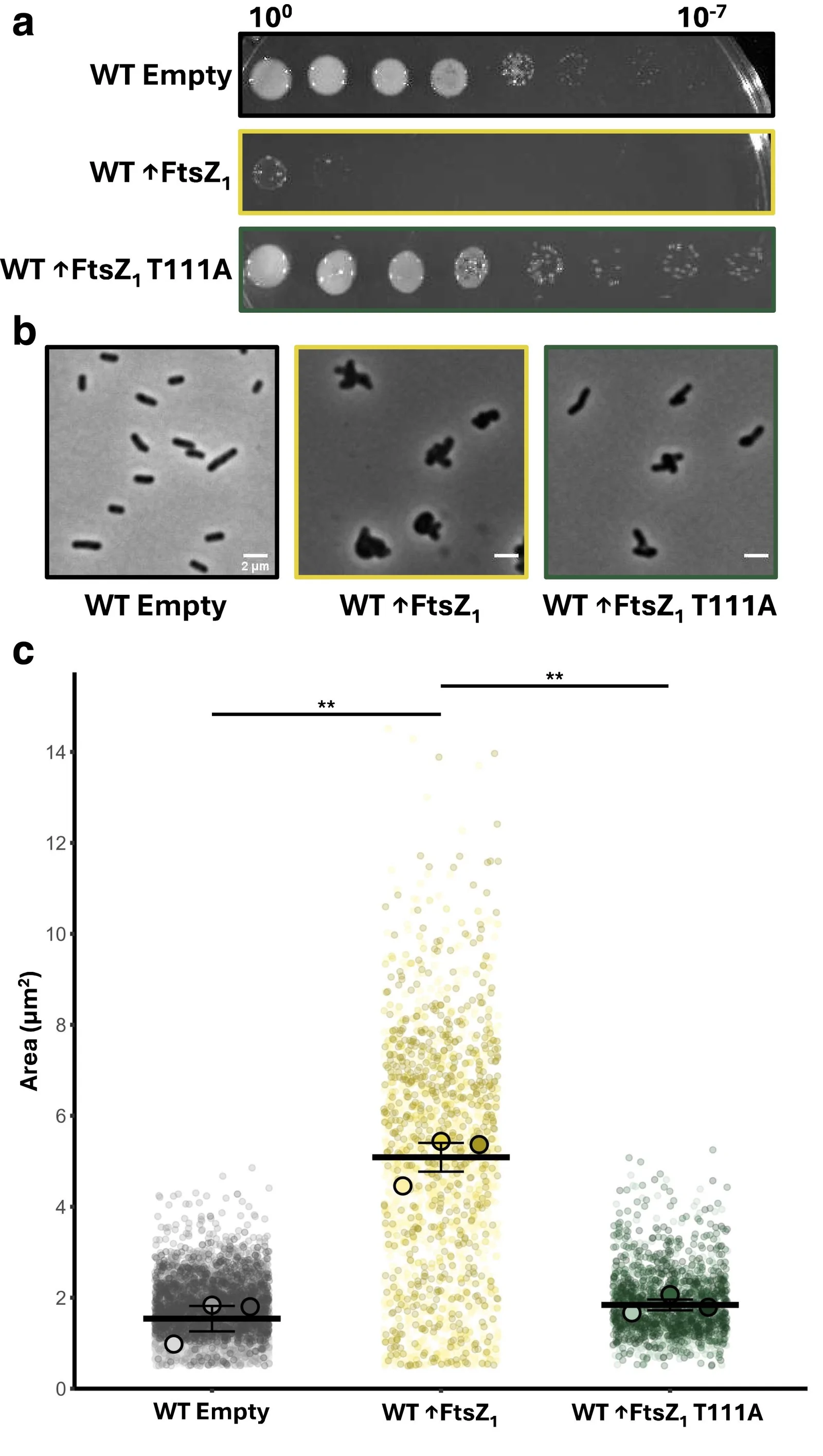
Expressing FtsZ_1_ results in a dramatic morphology and viability defect. a) Spotting assays of *A. tumefaciens* in the wild-type background with pSRKKM Ptac: empty vector (Empty), FtsZ_1_ and FtsZ_1_ T111A. When grown on ATGN Kan300 µg/mL 1 mM IPTG, the protein listed to the left is expressed from the plasmid. b) Phase contrast microscopy of *A. tumefaciens* in the wild-type background with pSRKKM Ptac: Empty, FtsZ_1_, and FtsZ_1_ T111A after 16 h of induction. c) Area measurements (µm^2^) after expressing the Empty Vector, FtsZ_1_, or FtsZ_1_ T111A for 16 h (*n* = 5973, 1933, 1665). Solid black lines indicate the average mean across biological replicates while large, colored circles indicate the mean for an individual biological replicate as described for cell biology measurements in Lord et al. 2020. Comparisons were conducted via Welch's 1-way ANOVA test and a Games–Howell post hoc test. *P*-values are represented by *P < 0.05 and **P < 0.01.

Because duplicate FtsZ GTPases might regulate the essential activity of FtsZ_AT_ during division, we used quantitative image analysis to assess morphological changes 16 h after the induction of FtsZ_1_ production (Fig. 4b and c). Plasmid-borne FtsZ_1_ production resulted in dramatic changes in morphology. Cells producing FtsZ_1_ lost their rod-shaped morphology and appeared to swell from the mid-cell while also forming short ectopic poles (Fig. 4b). Since conservation of the GTP-binding pocket among duplicate GTPase domains within the Hyphomicrobiales is a shared feature (Fig. 3c to e), we generated a GTPase-null variant of FtsZ_1_. The FtsZ T108A mutant in *E. coli* impairs Z-ring stability and constriction and results in a dramatically reduced GTP hydrolysis rate (Lu et al. 2001). We produced the equivalent T111A variant of FtsZ_1_ (Supplementary Fig. 6) and observed that most of the cells retained proper rod-shaped morphology (Fig. 4b). While some cells with intermediate phenotypes were observed, the average area across 3 biological replicates was consistent with an empty vector control (Fig. 4c; Supplementary Fig. 7, Supplementary File 1). Overall, these results suggest that the impairment of FtsZ_1_ GTPase activity reduces the toxic effects associated with FtsZ_1_ accumulation.

Notably, the mid-cell swelling phenotype observed when FtsZ_1_ is produced is not consistent with a total inhibition of FtsZ_AT_ activity in *A. tumefaciens*. The depletion of *ftsZ_AT_* prevents the establishment of FtsZ rings and termination of polar growth, and as a result, growth-active poles undergo tip-splitting events leading to the formation of elongated cells that accumulate active growth poles and, ultimately, lyse (Howell et al. 2019). The phenotype caused by FtsZ_1_ production also differs from the loss of FzlA in *A. tumefaciens,* which has also been shown to regulate septal peptidoglycan. Upon depletion of *fzlA*, *A. tumefaciens* cells form longer ectopic poles that look distinctly different from those produced when FtsZ_1_ accumulates (Payne et al. 2024). The distinct phenotype associated with FtsZ_1_ production suggests that excess FtsZ_1_ blocks cell division and inhibits polar growth in a way that is unique from the loss of either FtsZ_AT_ or FzlA. Furthermore, while duplicate FtsZ GTPase domains conserve MipZ interaction sites, with MipZ being conserved across the Alphaproteobacterial class, *A. tumefaciens* lacks a MipZ homolog (Supplementary Fig. 3, Supplementary File 1; Thanbichler and Shapiro 2006; Ehrle et al. 2017; Toro-Nahuelpan et al. 2019; Corrales-Guerrero et al. 2022). Therefore, we cannot directly compare the phenotypes between FtsZ_1_ and MipZ mutants, but this suggests that the FtsZ_1_ phenotype is due to an alternative factor.

The phenotype associated with FtsZ_1_ production suggests that FtsZ rings form and polar growth terminates properly; however, the mid-cell bulges and short ectopic pole formation suggest that septal peptidoglycan synthesis is misregulated and polar growth may reinitiate despite the block in cell division (Supplementary Fig. 8, Supplementary File 1). Remarkably, a similar phenotype with ectopic poles emerging from mid-cell swelling was observed during depletion of *ftsA* and *ftsW*, and in the double deletion of *pbp3a* and *pbp3b*, consistent with the hypothesis that septal PG is impaired during FtsZ_1_ expression (Howell et al. 2019; Williams et al. 2021).

### Hyperactive FtsW partially bypasses the effects of FtsZ_1_ expression

We hypothesized that if FtsZ_1_ accumulation impairs septal peptidoglycan biosynthesis pathways, a hyperactive variant of FtsW would be protective. Hyperactive variants of FtsW rescue other strains with impaired septal peptidoglycan synthesis by bypassing a late checkpoint in division (Modell et al. 2014; Lariviere et al. 2019; Li et al. 2021; Attaibi and den Blaauwen 2022; Payne et al. 2024). Therefore, we tested if a hyperactive variant of FtsW (F137L), FtsW*, could rescue the toxicity caused by FtsZ_1_ production. Indeed, the presence of FtsW* restored the viability of cells producing FtsZ_1_ to that of the parent strain (Fig. 5a). Quantitative image analysis indicated that the presence of hyperactive FtsW* reduced the cell area in both the wild-type and FtsZ_1_-producing strains (Fig. 5b and c; Supplementary Fig. 8, Supplementary File 1), presumably by bypassing the activation requirements for the septal peptidoglycan synthase. Despite this rescue, morphological defects persisted in these cells. Image analysis of cells 16 h after induction of FtsZ_1_ production revealed that most cells producing FtsZ_1_ still exhibit swelling and ectopic pole formation despite restored viability (Fig. 5b; Supplementary Fig. 8, Supplementary File 1).

**Fig. 5.**
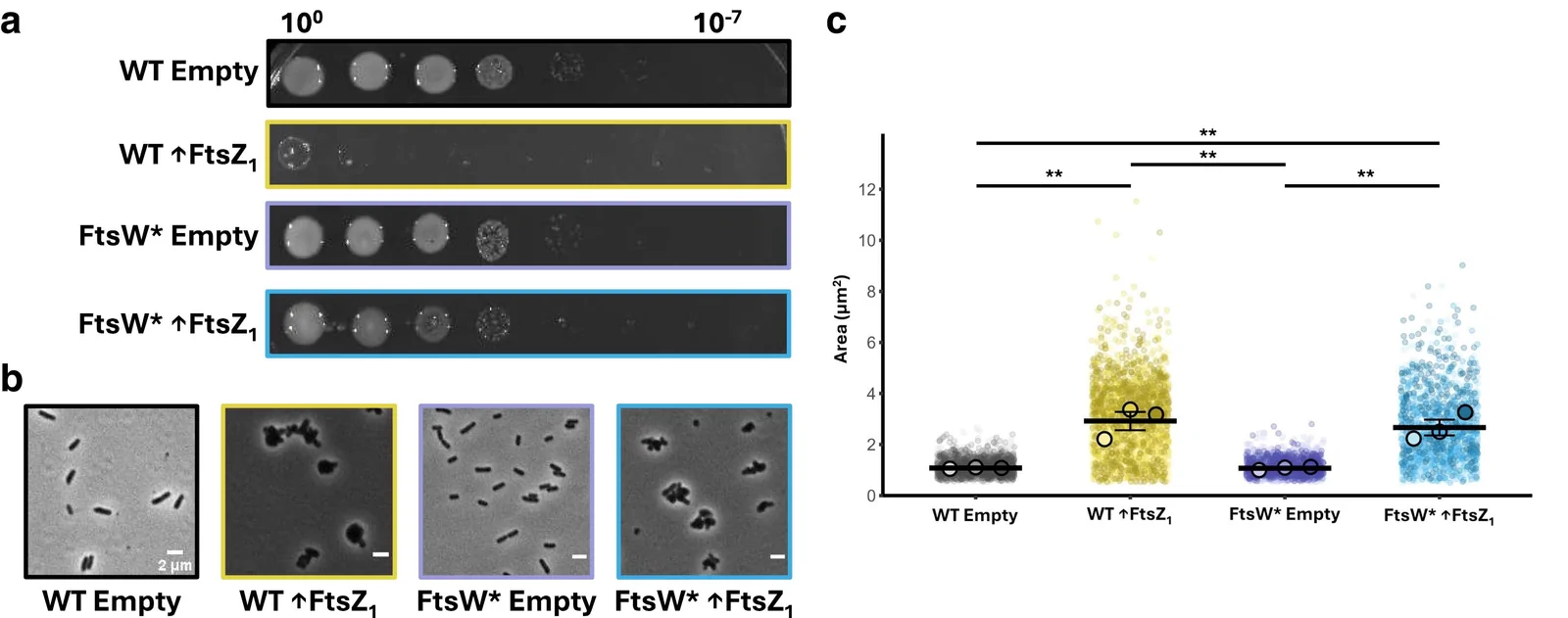
A hyperactive FtsW allele (FtsW*) partially resolves the phenotype of FtsZ_1_ expression. a) Spotting assays of *A. tumefaciens* in the wild-type background with pSRKKM Ptac: empty vector (Empty) and FtsZ_1_ as well as the FtsW* background with pSRKKM Ptac: Empty and FtsZ_1_. When grown on ATGN Kan300 µg/mL 1 mM IPTG, the protein listed to the left is expressed from the plasmid. b) Phase contrast microscopy of strains from previous panel after 16 h of induction. c) Area measurements (µm^2^) after 16 h of induction (*n* = 3302, 3108, 3110, 1947). Solid black lines indicate the average mean across 3 biological replicates while large, colored circles indicate the mean for an individual biological replicate as described for cell biology measurements in Lord et al. 2020. Comparing each mean, via 2-way ANOVA test with pairwise comparisons using estimated marginal means with Holm-adjusted *P*-values. *P*-values are represented by *P < 0.05, **P < 0.01, ***P < 0.001, and ****P < 0.0001.

The observation that pleomorphic cells have wild-type-like viability during FtsZ_1_ production is counterintuitive. Thus, we used time-lapse microscopy to better understand this complex phenotype and determine how these cells maintain viability when FtsZ_1_ was produced in the presence of FtsW*. For this experiment, instead of growing the cells for 16 h in liquid culture with inducer to drive FtsZ_1_ production, cells were continuously induced on agarose pads and imaged over time to capture morphological and division defects in real time. Consistent wild-type rod-shaped morphology and division events occurred in cells containing the empty vector over 12 h of observation (Fig. 6a, black box). Wild-type cells producing FtsZ_1_ were inhibited in cell division within 2 h and mid-cell swelling and/or ectopic pole formation was evident within 4 h, leading to loss of proper rod-shaped morphology (Fig. 6a, yellow box). To better understand this phenotype, cells were labeled with the fluorescent dipeptide HADA-DA for 5 h and 45 min to fully label the peptidoglycan and then imaged followed by a 15-min chase to visualize regions of peptidoglycan synthesis (Williams et al. 2021). In the absence of FtsZ_1_ production, unlabeled cell material was present at the cell poles and mid-cell (Fig. 6b, white arrowheads), consistent with proper peptidoglycan biosynthesis patterns in polar-growing *A. tumefaciens* cells. When FtsZ_1_ was induced, cells had large regions of decreased HADA-DA signal along the sidewalls adjacent to ectopic poles, but no mid-cell clearance was observed (Fig. 6b, yellow box). This observation suggests that the existing labeled peptidoglycan in the growth-pole-containing compartment became dispersed. This could indicate that peptidoglycan hydrolysis or remodeling occurs throughout this compartment in the absence of synthesis or that peptidoglycan biosynthesis is mislocalized. The absence of unlabeled regions at the growth pole or mid-cells is consistent with misregulation of peptidoglycan synthesis.

**Fig. 6.**
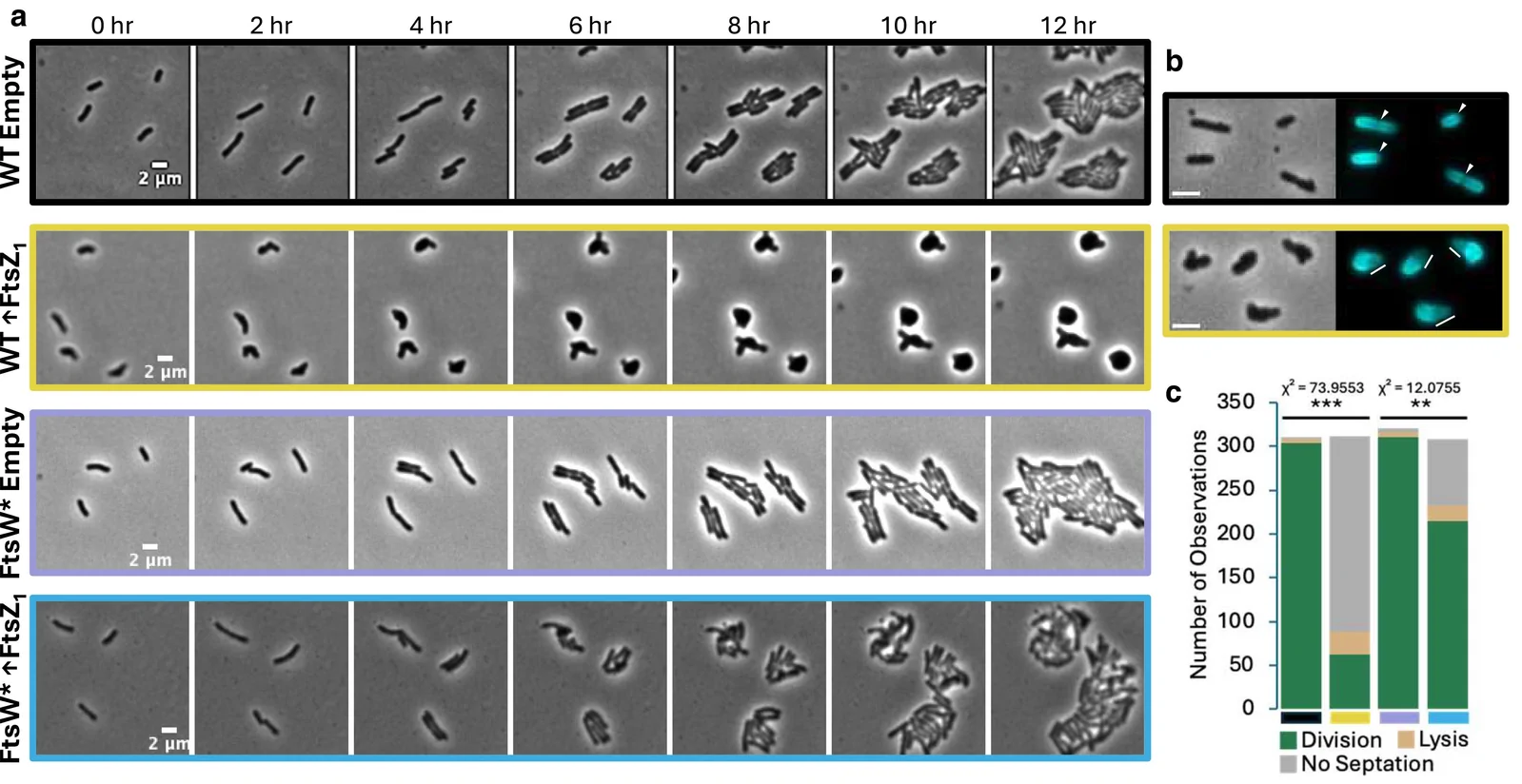
FtsZ_1_ inhibits the activation of septal peptidoglycan synthases which blocks septation. a) Phase contrast time-lapse microscopy of indicated *A. tumefaciens* strains harboring empty pSRKKM-P*_tac_* or pSRKKM-P*_tac_-ftsZ_1_*. Growth and division of individual cells in the presence of IPTG for 12 h is shown. b) The indicated strain was grown in the presence of the fluorescent dipeptide HADA-DA for 5 h and 45 min with IPTG to fully label the peptidoglycan. Following a brief chase of 15 min, wild-type cells harboring empty pSRKKM-P*_tac_* have narrow zones of unlabeled peptidoglycan at the poles and mid-cell (white arrowheads). In contrast, the expression of FtsZ_1_ results in larger regions of decreased HADA-DA signal (white bars). c) Classification of single cell fates observed through time-lapse microscopy for each strain as indicated by the colored bars. Fields of cells were imaged every 10 min for 16 h. The total number of cells observed was similar for all strains (*n* = 310, 311, 320, 308). Each cell was classified as dividing, failing to septate, or lysing. Differences between the numbers of observations were assessed using a χ² test. ** indicates *P* < 0.005; *** indicates *P* < 0.0005.

Notably, the FtsW* background restored cell division events in the presence of FtsZ_1_ production for most cells up to 4 to 6 h (Fig. 6a, blue box). We used the time-lapse data to quantify the frequency of cell septation, division, and lysis in each strain (Fig. 6c). When FtsZ_1_ was produced in a wild-type background, only ∼20% of cells divided; however, in the FtsW* background, ∼70% of cells divided (Fig. 6c). These results are consistent with our assessment of whole populations after 16 h of FtsZ_1_ induction and further support the claim that the presence of FtsZ_1_ results in a block in cell division that can be overcome with the hyperactive FtsW* background. FtsZ_1_ expression in the FtsW* background resulted in a fainter band compared to expression in the wild-type cells (Supplementary Fig. 6, Supplementary File 1). This difference may be a consequence of Z-ring turnover through successful cell-cycle progression, but it is possible FtsZ_1_ is produced at high enough levels to impair cell morphology but not to inhibit cell division.

Interestingly, when grown on the agarose pads for this time-lapse experiment, the FtsW* background partially rescued the cell morphology of cells producing FtsZ_1_, with most cells retaining proper rod-shaped morphology with some mid-cell swelling. This discrepancy led us to speculate that decreased cell wall integrity could be responsible for the persistent morphological differences observed after 16 h of FtsZ_1_ production in the FtsW* background in liquid culture (Fig. 5b). When grown in a liquid medium, bacteria encounter more direct and immediate osmotic challenges, which are known to change morphology and growth rates (Rojas and Huang. 2018; Ye et al. 2018). Thus, it is possible that osmotic differences exacerbate cell envelope mutant phenotypes when grown in liquid or on agarose or agar (Fig. 5; Fig. 6). In agreement, recent studies exploring single-cell measurements have also noted phenotypic discrepancies between growth in liquid and on solid media (Moffitt et al. 2012; Figueroa-Cuilan et al. 2021; Sun et al. 2021). Therefore, the FtsW* strain experiences different stresses when induced for FtsZ_1_ production for 16 h in liquid medium versus on agarose pads. Overall, these data suggest that incorporation of FtsZ_1_ into the Z-ring likely impairs septal peptidoglycan synthesis in *A. tumefaciens*, leading to decreased cell wall integrity and susceptibility to osmotic pressure. Future work will be necessary to understand the molecular mechanisms underpinning the influence of FtsZ_1_ on cell division more broadly in the Hyphomicrobiales.

### How could duplicate FtsZ GTPases regulate septal peptidoglycan activation?

Two independent FtsZ duplication events produced secondary FtsZ GTPases that have been maintained primarily in groups with a plant-associated lifestyle within the Hyphomicrobiales order. While the deletion of FtsZ_1_ in *A. tumefaciens* was not detrimental in an array of conditions, continuous production led to misregulation of septal peptidoglycan biosynthesis and lysis. We hypothesize that FtsZ_1_, the *A. tumefaciens* ortholog of the duplicate FtsZ GTPases in the *Rhizobiaceae* and *Phyllobacteriaceae* families, functions as a nonessential inhibitor of cell division, which may be conditionally beneficial during microbe–plant interactions. Given the pattern of conservation, it is likely that it serves a similar role in regulating division among other family members.

Across the bacterial domain, the ability of Z-ring to influence the activity of the septal peptidoglycan machinery FtsWIQLB is transduced through Z-ring anchoring proteins such as FtsA (Park et al. 2021; Santiago-Collazo et al. 2024). In general, the proper temporal assembly of the divisome complex serves as a checkpoint for the septum synthesis and ultimately cell separation. More specifically, FtsN has been shown to both interact with FtsA and activate the late divisome septal peptidoglycan synthases FtsW/FtsI (Addinall et al. 1997; Gerding et al. 2009; Park et al. 2021). A duplicate FtsZ could negatively impact septal synthase activation by directly or indirectly interfering with this checkpoint. The hyperactive FtsW* allele bypassed this effect because it can polymerize glycan strands without proper activation (Modell et al. 2014; Meeske et al. 2016; Rohs et al. 2018). Together, the observations that FtsZ_1_ production inhibits division and that an FtsW* allele can bypass this phenotype suggest that FtsZ_1_ may disrupt late divisome activation.

Since *A. tumefaciens* belongs to the *Rhizobiaceae* family, it is possible that one or more of the colored regions in Fig. 3c to e are required for its regulatory function. If the shared yellow region is implicated, the *Bradyrhizobium* duplicate may also influence cell division in a parallel mechanism. We hypothesize that these surface-exposed regions may serve as interfaces for protein–protein interactions. Future efforts will focus on the identification of FtsZ duplicate binding partners. FtsZ duplicates have already been shown to have regulatory roles in cyanobacteria and chloroplasts, where an FtsZ paralog functions to destabilize the Z-ring in a GTP/GDP ratio-dependent manner (Porter et al. 2021, 2023). Recently, Cao et al. demonstrated that differences in the GTPase domains allowed the duplicates to reduce Z-ring stability in *Arabidopsis thaliana* chloroplasts (Cao et al. 2025). Their work highlights how a duplicate FtsZ's ability to increase the turnover of the Z-ring can impact septal synthase activation. As described by Hu et al., faster Z-ring assembly increases the likelihood of FtsN and FtsWIQLB interaction: therefore, activating septal peptidoglycan synthesis (Hu et al. 2025). Meanwhile, destabilizing the Z-ring would drive fewer interactions between FtsN and FtsWIQLB. This mechanism is different from what we propose for the FtsZ duplicates in the Hyphomicrobiales, as previous in vitro work with *A. tumefaciens* proteins suggests that FtsZ_1_ does not affect Z-ring structure or turnover (Howell et al. 2019). While Howell et al. did not observe differences in structure or turnover, they only looked at FtsZ_AT_:FtsZ_1_ in a 1:1 ratio; therefore, it is possible that different stoichiometries could alter the dynamics. In this paper, we describe conserved amino acid sequences in the duplicate FtsZ GTPase domains that could scaffold protein–protein interactions. While further work is needed to discern if these sites drive or block specific interactions within the Z-ring, it is tempting to hypothesize that they function to disrupt late divisome recruitment, which is not yet well understood in the Hyphomicrobiales. Moreover, the putative FtsN ortholog, RgsS, in the *Phyllobacteriaceae/Rhizobiaceae* contains an extended cytoplasmic intrinsically disordered region and may have diverged in function and interacting partners from canonical FtsN (Krol et al. 2020). The differences in RgsS domain structure between the *Phyllobacteriaceae/Rhizobiaceae* and *Bradyrhizobiaceae* families (Krol et al. 2020), coupled with distinct regions of the duplicate FtsZ GTPase, may indicate that different binding partners activate FtsN/RgsS in the Hyphomicrobiales.

### Concluding remarks

We are fascinated by the conservation of duplicate FtsZ GTPases in plant-associated members of the Hyphomicrobiales. Plants often ward off potential infectious bacterial agents by secreting defensin-like peptides. Sublethal levels of these peptides have been shown to specifically block cell division and antagonize Z-ring function in *S. meliloti* (Li et al. 2002; Wu et al. 2012; Penterman et al. 2014; Heckel et al. 2014). The ChvGI 2-component system, which has historically been associated with host invasion, has been more recently shown to be activated by the disruption of cell division and to provide protection against antibiotics and antimicrobial peptides (Quintero-Yanes et al. 2022; Williams et al. 2022a, 2022b; Bouchier et al. 2025). Therefore, plant-associated lifestyles appear to coincide with adaptations that allow bacteria to modulate their growth in response to plant defenses. Perhaps, conditional expression of *ftsZ_1_* may modulate heteropolymeric FtsZ rings, leading to an inhibition or slowing of bacterial cell division, thereby giving plant-associated bacteria time to counteract environmental stressors during host invasion, rhizosphere association, or other niche-specific interactions. Furthermore, the regulation of division via modulation of Z-rings may provide a dynamic mechanism to tune division rates in changing environments, including during infection. Further research is needed to tease apart the complex relationship between expression, protein–protein interaction, and downstream functions of the FtsZ GTPase duplications.

## Supplementary Material

jkag275_Supplementary_Data

## Data Availability

Strains and plasmids are available upon request. The authors affirm that all data necessary for confirming the conclusions of the article are present within the article, figures, and supplemental files. Supplemental material available at G3 online.
