# Supplementary material for: Ancient truncated FtsZ paralogs likely tune cell division in Hyphomicrobiales
Source: G3 (Bethesda). 2026 Sep 26;16(11):jkag275. doi: 10.1093/g3journal/jkag275 (PMC13732431; doi:10.1093/g3journal/jkag275)
Supplement: Supplementary file 1 [file jkag275_supplementary_data.zip › FileS1.pdf]

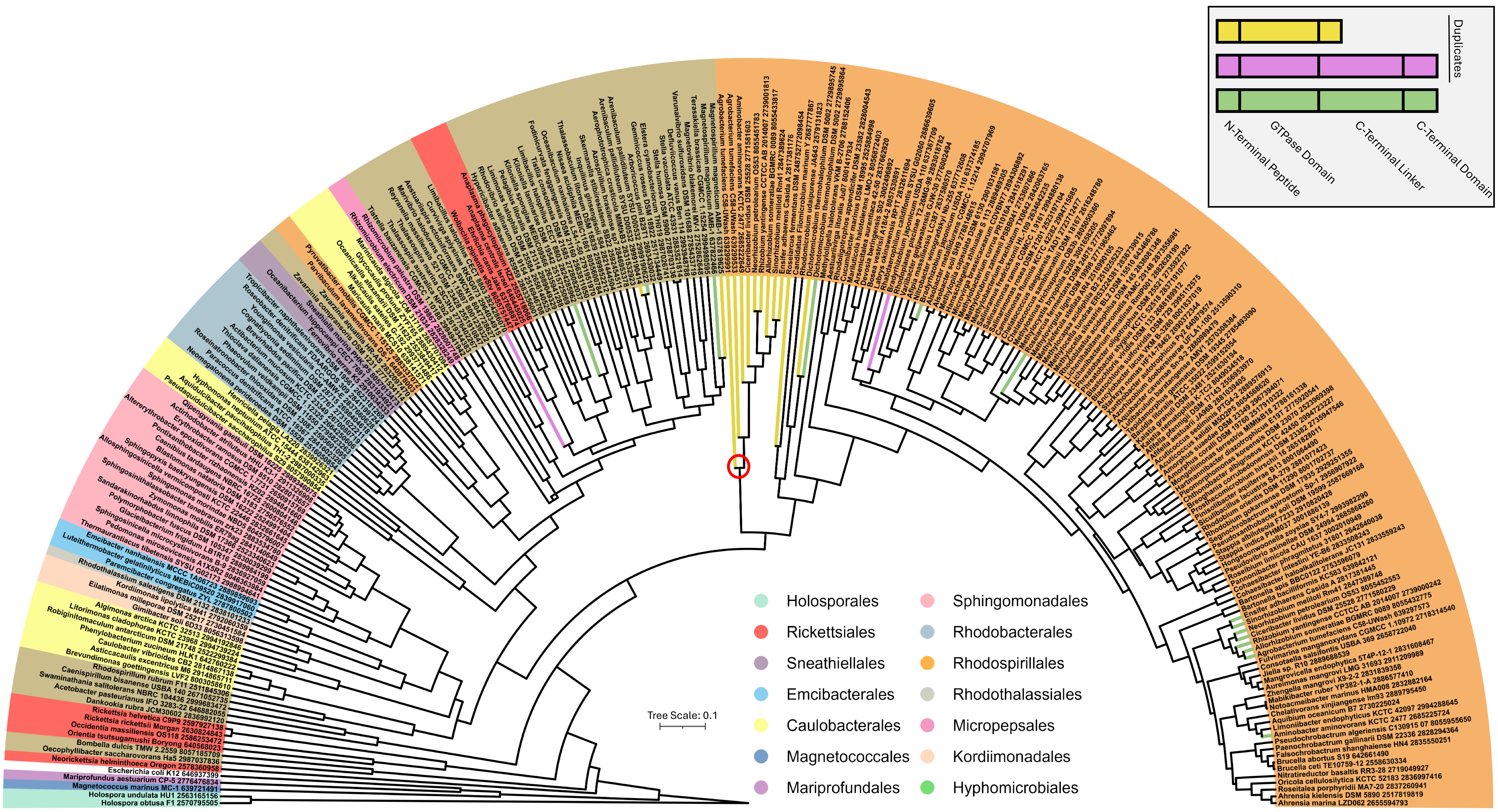

Figure S1. FtsZ duplications have arisen multiple times in the Alphaproteobacterial class. Branch length values were preserved along with original node orientation. Branches in black indicate that species that have a single copy of FtsZ. Colored branches indicate species with FtsZ duplications. Green branches lead to canonical FtsZ while yellow and purple branches indicate truncated and full-length duplicated FtsZ paralogs, respectively. Representative domain structures for canonical (green) and duplicate (yellow & purple) FtsZ copies are shown in the grey box in the top-right corner. Red circle indicates a node comprised solely of duplicated FtsZ paralogs. Color coded names indicates the Alphaproteobacterial order of the organisms containing the FtsZ protein.

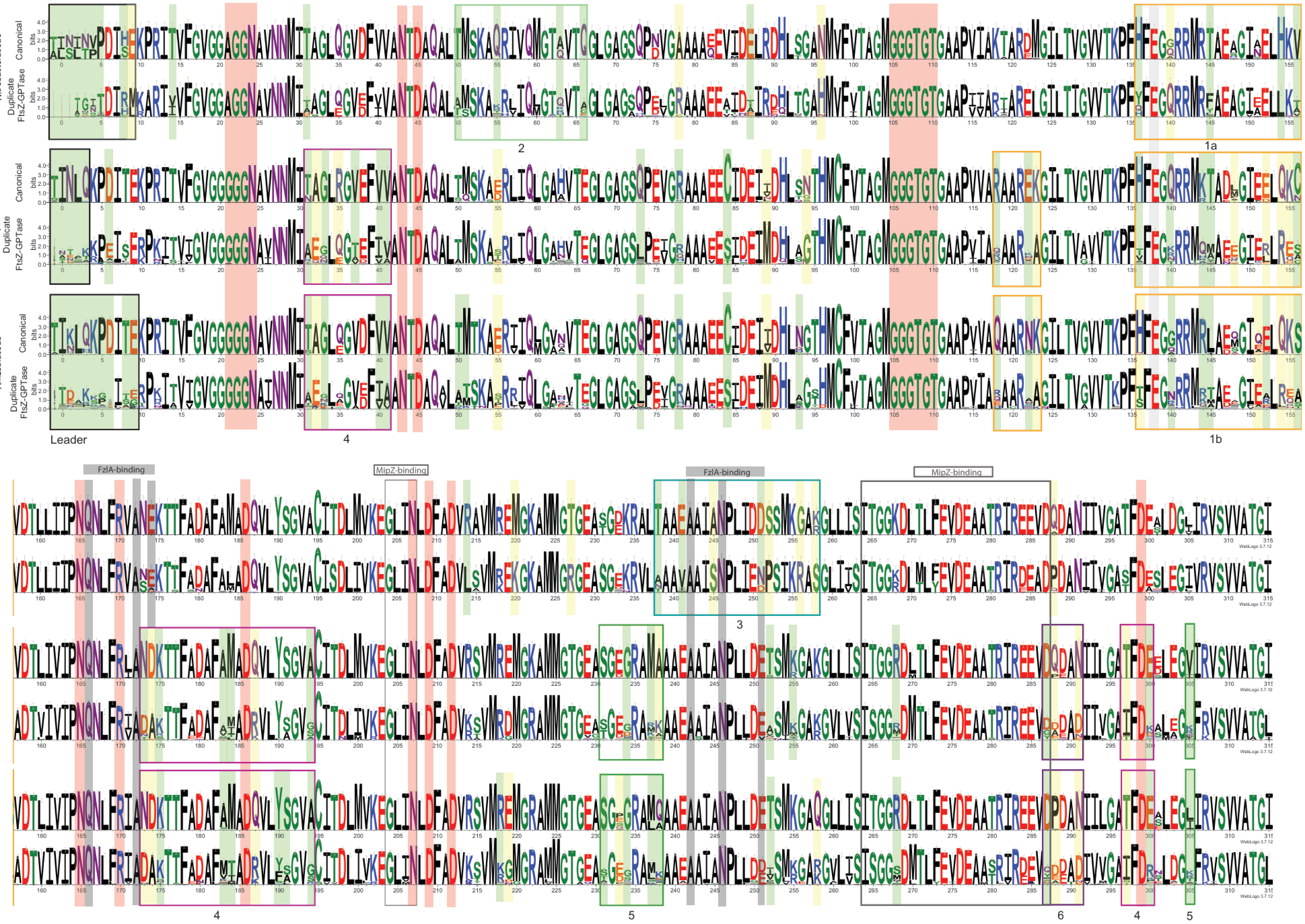

Figure S2. HMM logos comparing consensus sequences of canonical FtsZ to the duplicate FtsZ GTPase domain in each family. HMM logos of FtsZ GTPase and FtsZ GTPase duplications were made using WebLogo 3. Logos were constructed from protein sequences from *Nitrobacteraceae* (canonical n = 36, duplicate n = 38), *Phyllobacteriaceae* (canonical n = 80, duplicate n = 84), and *Rhizobiaceae* (canonical n = 142, duplicate n = 157) families. Amino acids are color coded according to chemical properties, with uncharged polar residues in green, neutral residues in purple, basic residues in blue, acidic residues in red, and hydrophobic residues in black. The height of each letter is proportional to the relative frequency of a given identity, and the height of the stack indicates the sequence conservation at that position. *E. coli* numbering is used for ease of comparison. Positions in which the duplicate FtsZ GTPase domain differs from canonical consensus have been highlighted green (loss of conservation) or yellow (conserved substitution with a chemical change). Residues important for GTPase activity are highlighted in red. Putative regulator-binding residues determined from alignments with *Caulobacter crescentus* FtsZ are marked in gray boxes for FzlA (Payne et al. 2024). and black-bordered boxes for MipZ (Corrales-Guerrero *et al*. 2022).

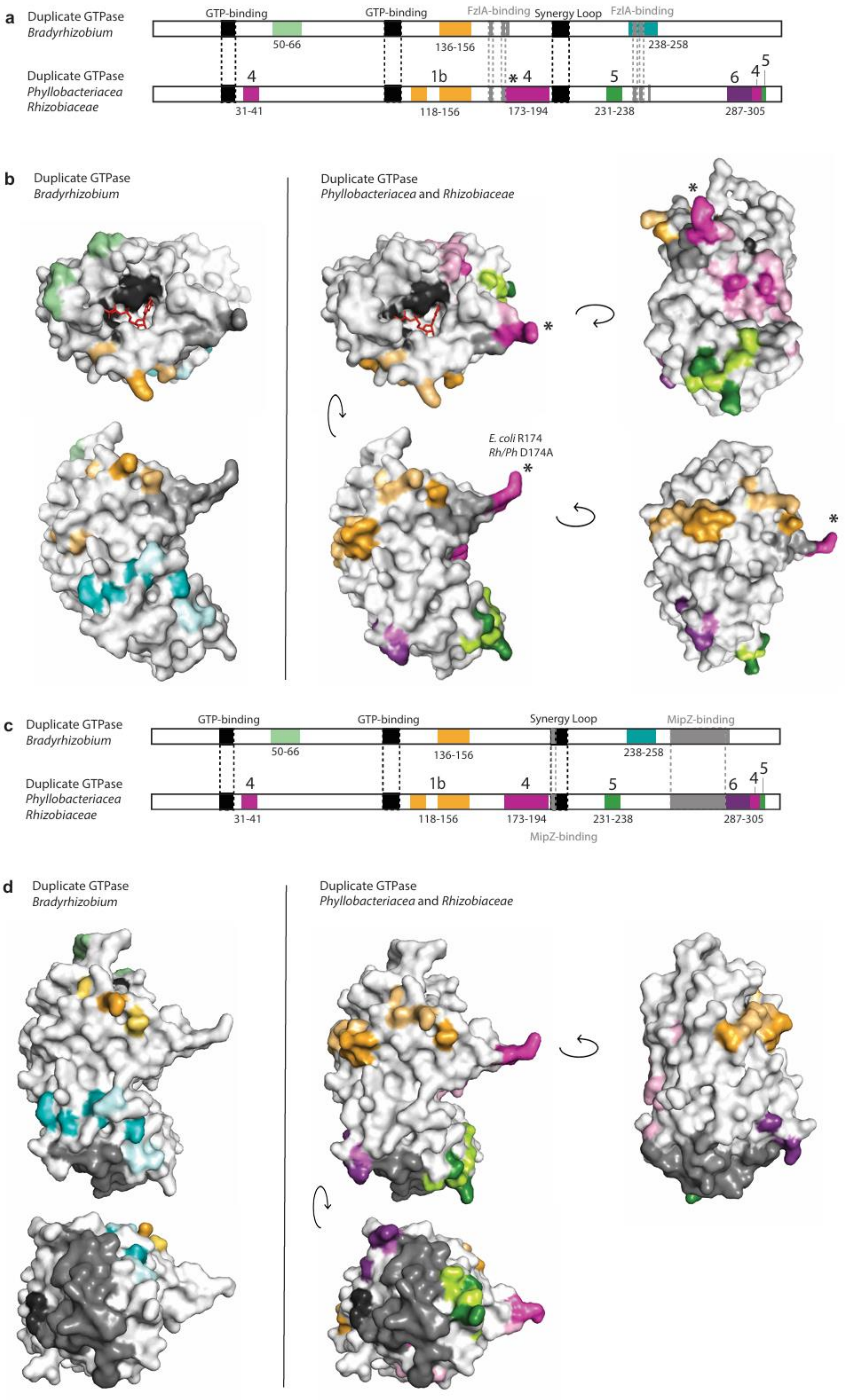

Figure S3. Regulator-interacting residues are conserved in the GTPase domain duplications. (a) and (b) Schematic summary of HMM analysis of the entire duplicated GTPase domains and structural models as shown in Figure 3 including putative FzlA residues determined from alignments with *Caulobacter crescentus* FtsZ marked in gray (Payne et al. 2024). Position R174 (*E. coli* numbering and identity) is marked in the *Ph/Rh* structural model as this position has undergone the substitution D184A in these families but is unlikely to protrude from the loop in the same way as R174. (c) and (d) Schematic summary of HMM analysis of the entire duplicated GTPase domains and structural models as shown in Figure 3 including putative MipZ residues determined from alignments with *Caulobacter crescentus* FtsZ marked in gray (Corrales-Guerrero *et al*. 2022).

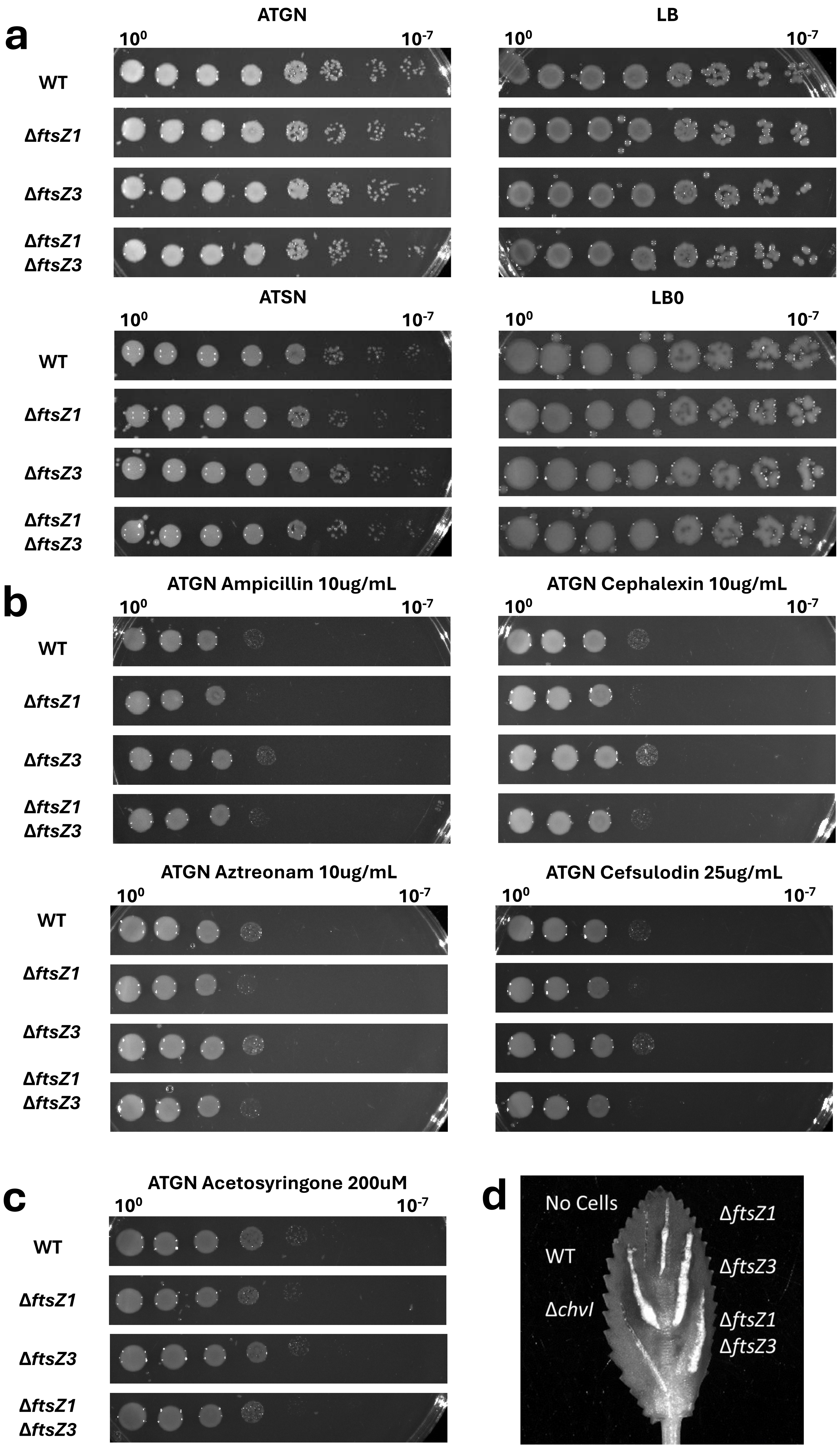

Figure S4. Deletion of *ftsZ*$_1$, *ftsZ*$_3$, or both does not impair survival in a wide array of growth conditions. We assessed growth and viability using spot titers on solid media, for WT and each deletion strain, across (a) standard laboratory conditions including ATGN, ATSN, LB, and LB lacking sodium chloride (LB0), (b) in the presence of sublethal cell-wall targeting antibiotics, (c) and in the presence of a plant signal that activates virulence. (d) Tumorigenesis was tested for WT and each deletion strain using a *Kalanchoe sp.* leaf assay.

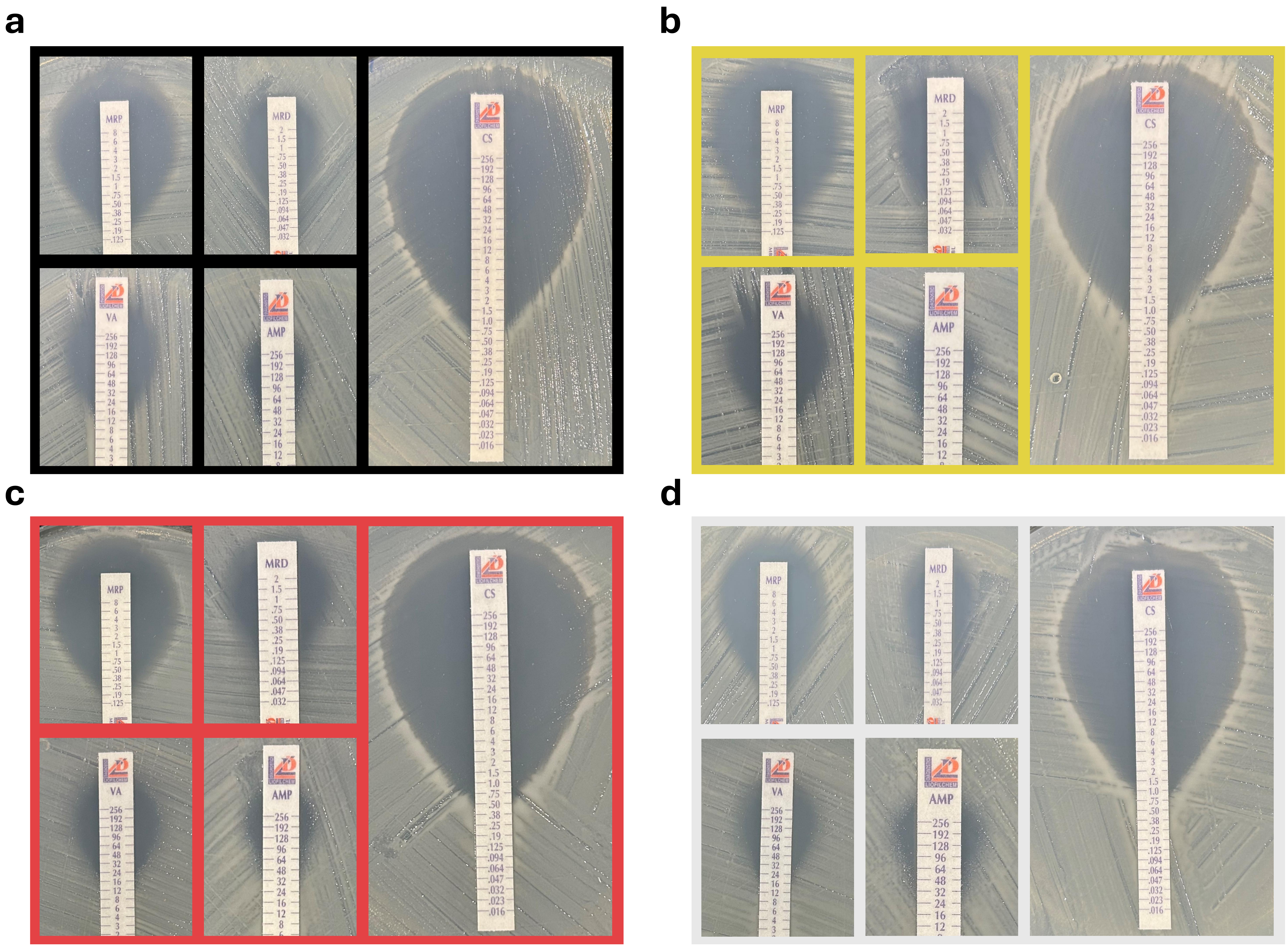

Figure S5. Deletion of $ftsZ_1$, $ftsZ_3$, or both does not impair minimal inhibitory concentrations of tested antibiotics. We assessed the minimal inhibitory concentrations across meropenem (MRP), meropenem + EDTA (MRD), vancomycin (VA), ampicillin (AMP), and colistin (CS), for (a) WT, (b) $\Delta ftsZ_1$, (c) $\Delta ftsZ_3$, and (d) the double deletion strains.

a

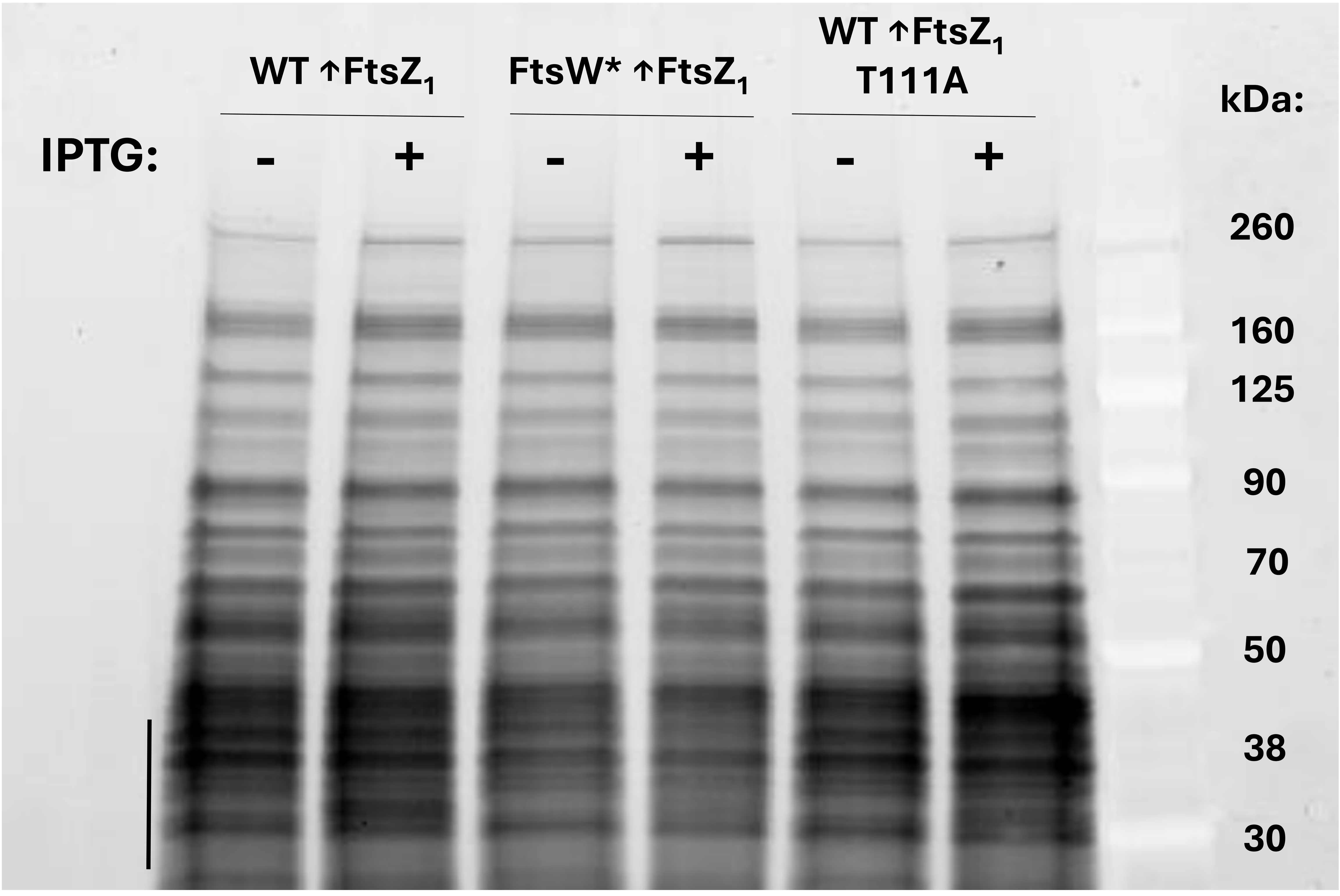

b

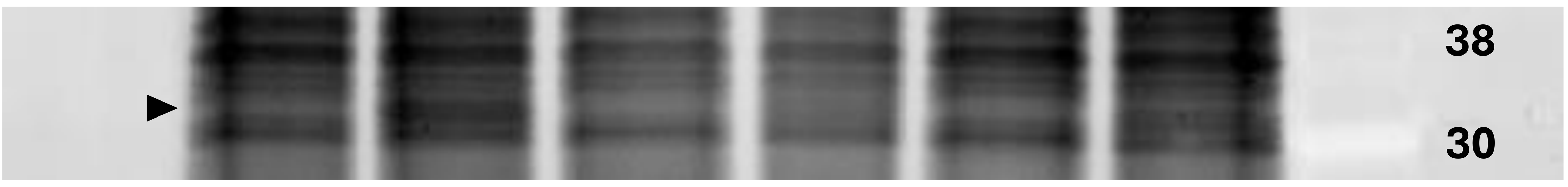

Figure S6. $FtsZ_1$ overexpression is present in differing amounts between WT ↑$FtsZ_1$, FtsW* ↑$FtsZ_1$, and WT ↑$FtsZ_1$ T111A observed through Sypro Ruby total protein-stained gel.
(a) Visualization of cell lysates, loaded to 10 µg per well, after 16 hours of growth in the presence or absence of IPTG. $FtsZ_1$ is predicted to be 32.91 kDa. Black bar to the left of lane 1 indicates cropped section for panel b. (b) Cropped gel to isolate protein between 30 and 38 kDa as indicated by ladder. Black arrowhead indicates size of expected $FtsZ_1$ band in overexpression conditions.

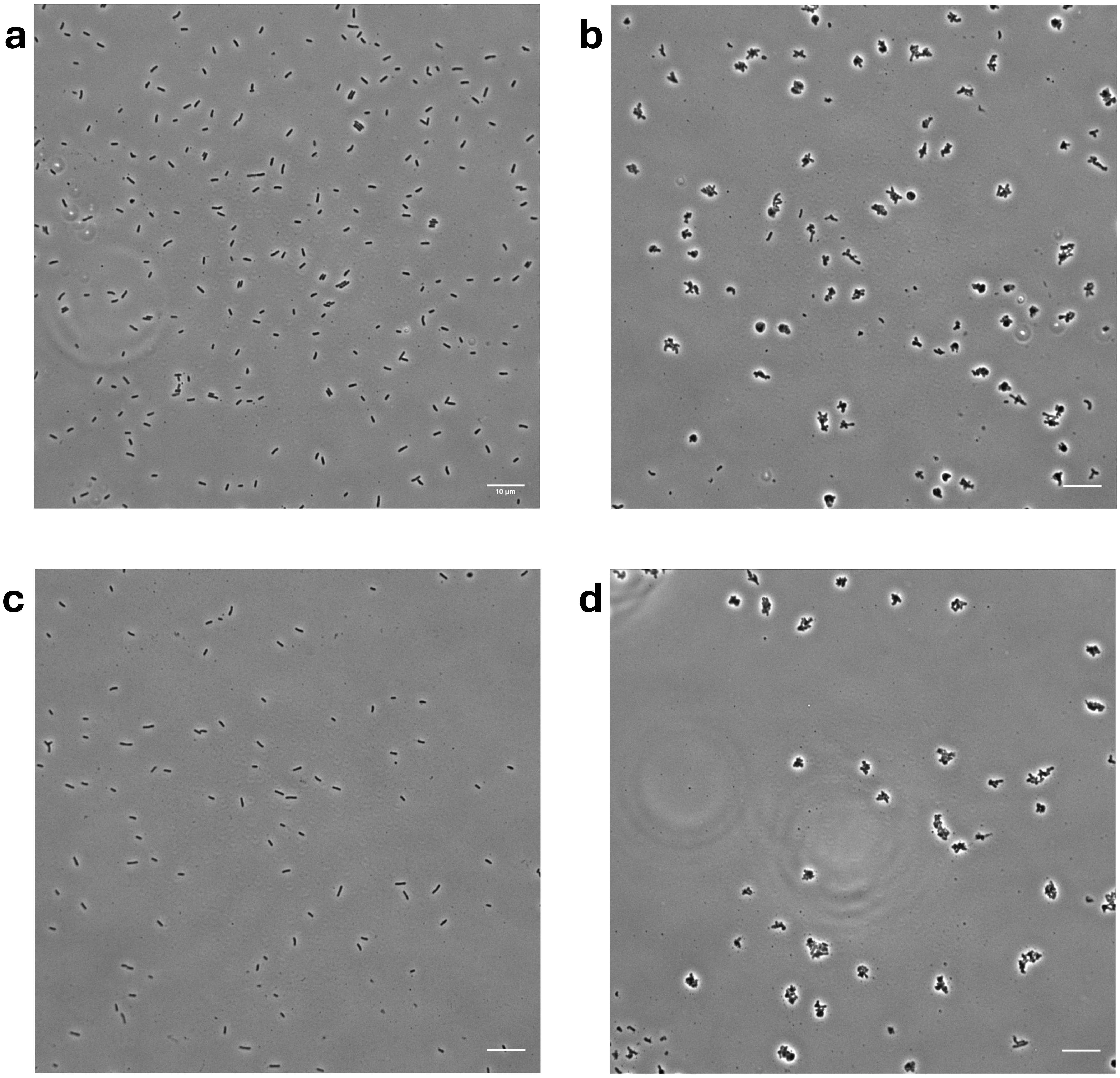

Figure S7. The overexpression of $FtsZ_1$ in the FtsW* background results in cell swelling and loss of rod-shaped morphology. Representative fields of view from phase contrast microscopy for (a) WT Empty Vector, (b) WT ↑$FtsZ_1$, (c) FtsW* Empty Vector, and (d) FtsW* ↑$FtsZ_1$. Scale bars are set to 10 µm.

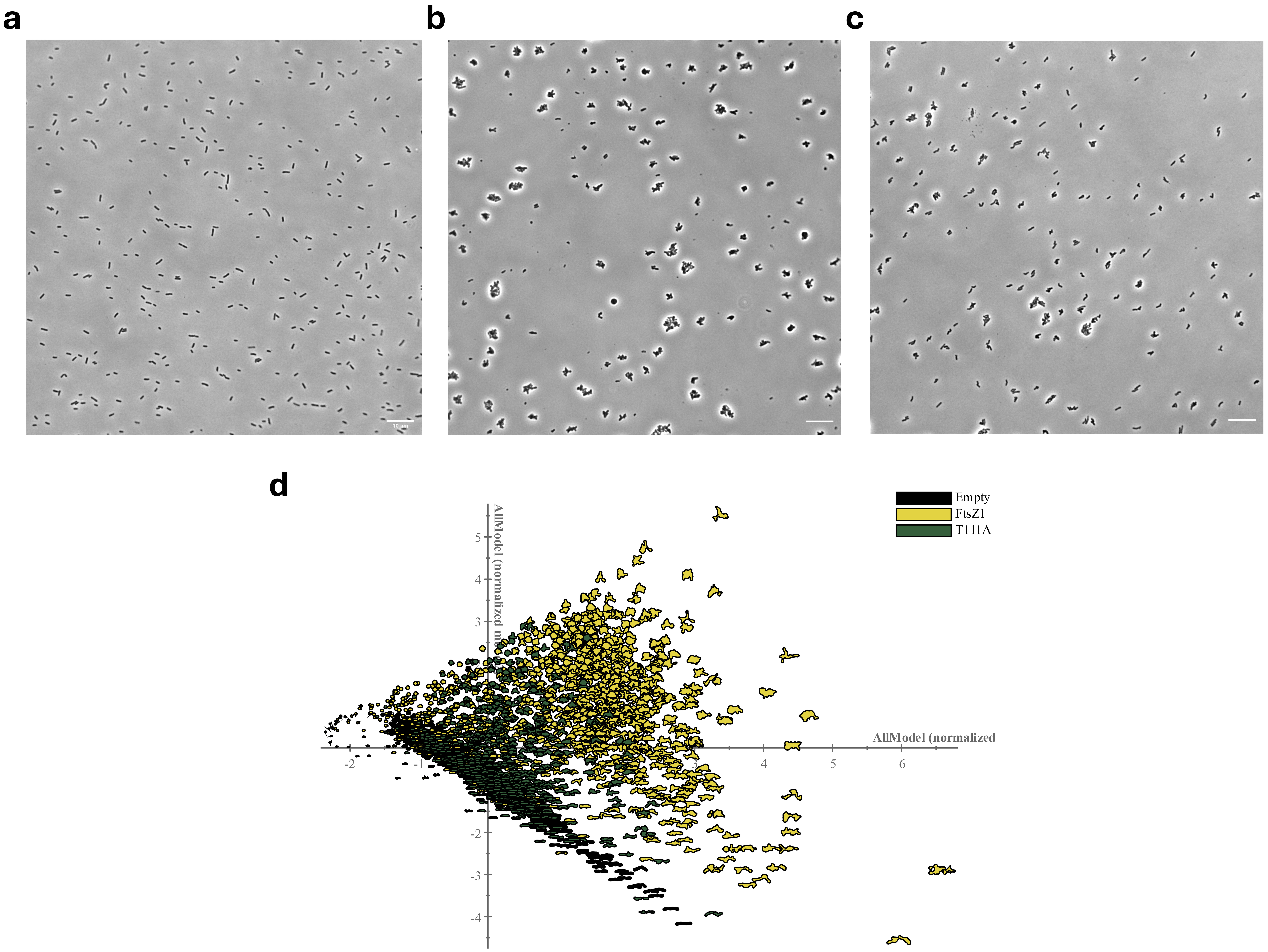

Figure S8. The overexpression of $FtsZ_1$ T111A results in reduced cell swelling phenotype compared to the overexpression of $FtsZ_1$. Representative fields of view from phase contrast microscopy for (a) WT Empty Vector, (b) WT $\uparrow FtsZ_1$, and (c) WT $\uparrow FtsZ_1$ T111A. Scale bars are set to 10 µm. (d) Principal component analysis, generated in CellTool, for one biological replicate of the strains shown above.
